# Oncologic Photodynamic Therapy: Basic Principles, Current Clinical Status and Future Directions

**DOI:** 10.3390/cancers9020019

**Published:** 2017-02-18

**Authors:** Demian van Straten, Vida Mashayekhi, Henriette S. de Bruijn, Sabrina Oliveira, Dominic J. Robinson

**Affiliations:** 1 Cell Biology, Department of Biology, Science Faculty, Utrecht University, Utrecht 3584 CH, The Netherlands; demianvanstraten@gmail.com (D.v.S.); v.mashayekhi@uu.nl (V.M.); s.oliveira@uu.nl (S.O.); 2 Center for Optical Diagnostics and Therapy, Department of Otolaryngology-Head and Neck Surgery, Erasmus Medical Center, Postbox 204, Rotterdam 3000 CA, The Netherlands; h.debruijn@erasmusmc.nl; 3 Pharmaceutics, Department of Pharmaceutical Sciences, Science Faculty, Utrecht University, Utrecht 3584 CG, The Netherlands

**Keywords:** photodynamic therapy, clinical trials, cancer, treatment outcome, preclinical, future

## Abstract

Photodynamic therapy (PDT) is a clinically approved cancer therapy, based on a photochemical reaction between a light activatable molecule or photosensitizer, light, and molecular oxygen. When these three harmless components are present together, reactive oxygen species are formed. These can directly damage cells and/or vasculature, and induce inflammatory and immune responses. PDT is a two-stage procedure, which starts with photosensitizer administration followed by a locally directed light exposure, with the aim of confined tumor destruction. Since its regulatory approval, over 30 years ago, PDT has been the subject of numerous studies and has proven to be an effective form of cancer therapy. This review provides an overview of the clinical trials conducted over the last 10 years, illustrating how PDT is applied in the clinic today. Furthermore, examples from ongoing clinical trials and the most recent preclinical studies are presented, to show the directions, in which PDT is headed, in the near and distant future. Despite the clinical success reported, PDT is still currently underutilized in the clinic. We also discuss the factors that hamper the exploration of this effective therapy and what should be changed to render it a more effective and more widely available option for patients.

## 1. Introduction

Photodynamic therapy (PDT) is based on a photochemical reaction between a light activatable molecule or photosensitizer (PS), light, usually in the visible spectrum, and molecular oxygen. These three components are harmless individually, but in combination result in the formation of reactive oxygen (ROS) species [1] that are able to directly induce cellular damage to organelles and cell membranes depending on where they are generated [2].

PDT is a two-stage procedure consisting of the intravenous, intraperitoneal or topical administration of a PS, or PS precursor, followed by an exposure to light. This two-stage procedure significantly reduces side effects, as the harmless PS is activated only via a directed illumination, resulting in local tissue destruction.

### 1.1. History of PDT

The history of PDT has been described extensively [1,3,4]. The therapeutic potential of light has been employed for thousands of years. Over 3000 years ago, ancient Egyptian, Chinese and Indian civilizations already used light in combination with reactive chemicals to treat conditions like vitiligo, psoriasis and skin cancer [3]. In 1900, the observations of two different researchers led to the discovery of cell death induced by a combination of chemicals and light. Working for Professor Hermann von Tappeiner, the German student Oscar Raab studied the effects of the dye acridine on Infusoria, a species of Paramecium. He observed that acridine toxicity varies depending on its exposure to light [5]. In the same year, the French neurologist, Jean Prime, found that orally administered eosin, used to treat epilepsy patients, induced dermatitis when exposed to sunlight [6]. Further investigation of Raab’s discoveries by von Tappeiner resulted in the new term “Photodynamic Action” [7]. The first application of this approach in humans was performed by Friedrich Meyer-Betz using a porphyrin found in haemoglobin, called haematoporphyrin. When applying it to his own skin, he observed pain and swelling on light exposed areas [8]. Later studies done by Lipson et al. [9] using a haematoporphyin derivative (HPD) showed that this compound accumulated in tumors and emitted fluorescence. These properties in combination with the decreased dosage compared to crude haematoporphin made it a useful diagnostic tool [9]. A decade later, Diamond et al. showed HPD could be used to treat cancer in mice and observed decreased glioma growth for several weeks after HPD treatment before the deeper tumor tissue begun to regrow [10]. The efforts of Dougherty et al. in the 1970’s paved the way for PDT as it is known today. First they observed complete mammary tumor eradication in mice using HPD in combination with red light [11]. A second study using 25 patients with skin cancer showed a complete response in 98 out of 113 tumors, partial response in 13 and only two tumors appeared PDT resistant [12]. These findings were pivotal in the first clinical approval for the treatment of bladder cancer, in Canada, in 1993.

### 1.2. Aims of This Review

Since its regulatory approval as a cancer therapy, PDT has been subject of numerous studies and has proven to be an effective form of cancer therapy. Despite its potential and the growing body of knowledge on this modality, it is underutilized in the clinic. This review provides an overview of oncologic PDT as it is applied in the clinic today. Clinical studies performed in the last ten years will be employed to illustrate the efforts made to tackle the current limitations of PDT in the clinic. Finally, examples from the most recent preclinical studies will be given to show in which directions PDT is headed, both in the near and distant future. The aims of this review will therefore be: to analyze the current state of PDT in the clinic and to give insights as to how the future of PDT will look like as a (first-line) treatment for cancer.

## 2. Principles of PDT

### 2.1. Photodynamic Reactions

**Figure 1:**
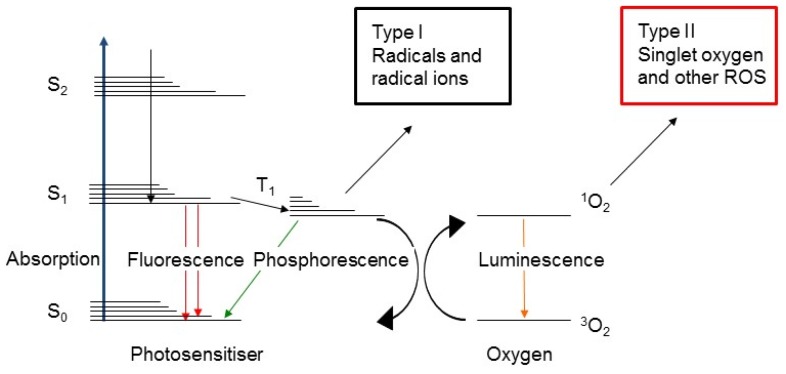
Schematic representation of type I and type II reactions following photosensitizer activation upon illumination.

Although the precise mechanism of action of PDT is an ongoing topic of investigation, its molecular effects are accepted to be based on the reaction of a light activated PS with other molecules, creating radicals [13]. Illumination of a PS leads to the absorption of a photon and promotes the PS to its excited singlet state, or S_1_, in which an electron is shifted towards a higher-energy orbital (Figure 1). From this unstable and typically short-lived state, the PS can return to its ground state S_0_ by converting its energy into heat or fluorescence, a feature which can be used for the purposes of diagnostics and optical monitoring [14]. Alternatively, intersystem crossing can occur resulting in the population of the PS as excited triplet state T_1_. In this T_1_ state, the PS can transfer its energy by phosphorescence or by colliding with other molecules to create chemically reactive species via two types of reactions [13,15]. T_1_ can react with a number of organic substrates or solvents and transfer an electron or a proton to form radical anion or cation species, respectively. Typically, the PS reacts with an electron donating substrate to form PS—that subsequently reacts with oxygen to form superoxide anion radicals. This is called a type I reaction. In a type II reaction, T_1_ reacts directly with ground state oxygen ^3^O_2_ by transfer of energy to form singlet oxygen ^1^O_2_ which is a highly reactive oxygen species (ROS) [15]. The exact molecular mechanisms of these photochemical reactions have been described in detail elsewhere [16].

The production of singlet oxygen and superoxide anions will result in cytotoxicity as both products can directly react with and damage biomolecules such as lipids, proteins and nucleic acids [2]. The superoxide anions formed in type I reactions are not particularly damaging in biological systems directly, but can be part of a reaction that produces hydrogen peroxide. When hydrogen peroxide reacts with superoxide anions via a Fenton reaction, very reactive hydroxyl radicals can be formed that are easily capable of adding to double bonds or abstract hydrogen atoms of nearly all biomolecules [17]. For instance, reacting with a fatty acid would form a hydroxylated product that is itself a radical, thereby initiating a chain reaction of lipid peroxidations, causing membrane damage.

Most PSs are thought to act through type II reactions where singlet oxygen is the main molecule causing oxidative cellular damage. The reaction of singlet oxygen with membrane lipids will result in lipid peroxidation and can lead to disruption of cellular membranes. It can also react with amino acids, which might impair the functionality of vital proteins. Since singlet oxygen is highly reactive, it’s lifetime is in the order of 40 ns and has a maximum action radius of about 20 nm. [18,19]. This short action radius (less than the diameter of most organelles) together with localized PS activation by only illuminating target tissues, theoretically renders PDT very specific and controllable. It also means the localization of the PS influences the site of action of PDT at the subcellular level [19].

### 2.2. PDT at a Cellular Level

The cellular response to photodamage is strongly dependent on multiple factors of which PS localization is the key [13]. The intracellular site of action is PS dependent and plays a significant part in the fate of the cell. In a study comparing the importance of PS subcellular location with chemical efficiency in inducing cell death, the PS cristal violet (CV) that was less efficient at producing radicals was equally efficient in inducing cell death as methylene blue (MB), that produced 10 times as much radicals. This is predominately due to the cytolocation of the PSs as MB localized towards the cytosol and lysosomes while CV ended up in mitochondria, suggesting localization is more important rather than the amount of radicals formed [20].

Depending on its characteristics (see later in this review) a PS will generally localize towards organelles such as the plasma membrane, lysosomes, mitochondria, Golgi apparatus or endoplasmic reticulum (ER) [21]. The cytoskeleton and cell adhesion components have also been described as PS targets [22]. Even though photodynamic action affects many targets, three main mechanisms of photodamage induced cell death have been described: apoptosis, necrosis and autophagy. The ability of PDT to activate multiple cell death pathways circumvents the problem of apoptosis resistant cells in tumours, which can be a major obstacle for other cancer therapeutics [23].

#### 2.2.1. Apoptosis

Apoptosis is a controlled mechanism of cell-death with highly regulated processes [23]. It can be initiated via numerous pathways following PDT-induced damage of several organelles [24]. PSs that localize to mitochondria are the most likely to induce apoptosis [25]. This is generally accepted because mitochondria play a key role in the majority of apoptotic pathways and it can be expected that damage to this organelle will lead to apoptosis [24]. Photodamage to mitochondria induces permeabilization of its membranes, which results in leakage of cytochrome c into the cytosol [26]. This, in turn, will activate the caspase mediated apoptotic pathway. However, numerous other apoptotic signal transduction pathways that are being activated upon photodamage have been described (reviewed extensively by [24,27,28]).

#### 2.2.2. Necrosis

With extensive damage to the cell, components of the apoptotic pathway may be damaged and apoptosis can’t be properly executed. With higher PDT-dosage depending on the amount of PS and light, increasing cell damage is observed leading to necrosis rather than apoptosis [29,30,31]. Necrosis is also more often observed when the PS site of action is the plasma membrane. When for instance Photofrin^®^ is activated in the cytoplasm, it induces apoptotic cell death. On the other hand, when it is activated in the plasma membrane by altering the incubation protocol, it induces more necrotic cell death [32]. In contrast with the apoptotic pathway, necrosis is considered less regulated. Photodamage to the plasma membrane results in leakage of intracellular material to the direct environment which can cause inflammation [33]. The role of inflammation and immune reactions during PDT will be discussed later in this review.

#### 2.2.3. Autophagy

A cell has the capability to recycle damaged organelles and cytoplasmic components by means of autophagy. The damaged particles are engulfed by a double membrane structure named autophagosome which fuses with lysosomes to degrade its contents [34]. Although it is considered a cytoprotective mechanism, autophagy has also been observed as a cell death mechanism in response to PDT [35,36]. When apoptosis is impaired, autophagy appears to be the main process responsible for cell death [37,38]. This also appears to be depended on PDT-dose as with lower doses (and less damage) autophagy functions as a protective mechanism while with higher PDT doses, autophagic cell-death can be initiated (reviewed in [39]). PS localization is also of importance as mitochondrial- and ER-targeted PS induce a prosurvival autophagic response [25], while lysosomal-targeted PS can inhibit autophagy [40].

Overall, determining the outcome of PDT on a cellular level is complex. Nevertheless, some general themes can be observed [28]. With high doses of PDT or PS localization to the plasma membrane, necrosis is the dominant form of cell-death. With mild PDT and damage to the mitochondria or anti-apoptotic components, apoptosis is triggered. With low PDT induced damage to organelles, autophagy is initiated to try and repair the damage. However, when the protective capacity of autophagy is overwhelmed or compromised due to for instance lysosomal damage, autophagy can induce cell death. Elucidating the exact effects of PSs and subsequent responses on a cellular level is crucial to understand the effect of PDT.

### 2.3. PDT at a Tumor Level

The phototoxic effect of PDT, as currently employed in the clinic, is in general not tumor cell selective. PSs are taken up by both healthy and tumor cells. In general, normal tissues are capable of eliminating or clearing the PS over time, while tumor tissues cannot, due to inexisting lymphatics. This leads to some rentention of PS in tumor tissue, which combined with the localized activation by specific illumination, gives PDT some selectivity. Factors that affect the preferential accumulation of PS towards tumor neoplastic tissue are complex and multiple theories exist based on several mechanisms [41]. A popular theory which applies to all PSs is based on the morphological differences between healthy and tumor tissues. Due to the rapid and uncontrolled growth of tumor cells, solid tumors have abnormal, unorganised vasculature with a defective inner lining. Consequently, tumor endothelium is leaky and macromolecules can extravasate into the extravascular space. Moreover, they are retained longer compared to healthy tissues due to the impaired lymphatic drainage in tumor tissue. This phenomenon is called the Enhanced Permeability and Retention (EPR) effect and is often utilized in cancer therapeutics [42]. Other theories attribute localization to increased expression of certain receptors on tumor cells, decreased intratumoral pH or tumor associated macrophages (TAM) that phagocytise PS molecules [43]. These theories are not fully understood and are heavily influenced by PS characteristics (hydrophobicity/-philicity), tumor type and dosage, amongst other aspects [44].

PDT is considered to have three main mechanisms of tumor destruction. Due to the localization and activation of the PS inside tumor tissue, ROS generated can directly kill the malignant tumor cells. Secondly, PDT can target tumor vasculature thereby compromising the supply of oxygen and essential nutrients. The third mechanism is the PDT activated immune system, inducing an inflammatory and immune response against tumor cells.

#### 2.3.1. Direct ROS Effects

Just as the subcellular PDT site of action is important for the fate of the affected cell, cellular site of action is important for the fate of the tumor. Like healthy tissues, solid tumors can be divided in distinct tissues with different cell types. Tumors are made up of the parenchyma consisting of the malignant cells and the stroma, which is the supportive, vascularised tissue. Tumor stroma includes plasma protein-rich interstitial fluid, structural proteins, fixed connective tissue cells and inflammatory cells and can make up as much as 90% of the tumor mass [45]. All solid tumors require stroma to grow since it supports the blood vessels that provide nutrients and oxygen and regulate waste disposal [46]. One can imagine different PDT outcomes depending on what part of the tumor is affected. The most direct form of tumor damage done by PDT is the killing of parenchyma cells. PSs that accumulate in tumor parenchyma have the obvious effect of cell damage and subsequent tumor cell apoptosis or necrosis. However, early studies already showed that direct destruction of neoplastic cells is not enough for tumor cure [13]. This led to the belief that damage to tumor stroma plays a major role in PDT efficacy, a hypothesis also recognized in other fields of cancer therapeutics [47]. The interactions between tumors and their extracellular matrix play a critical role in tumor cell growth, motility and invasiveness indicating the potential effect of modulating these interactions [48,49]. PDT induced damage to structural proteins such as integrins could disrupt essential stromal-tumor signalling [48]. Also, the destruction of stromal fibroblasts inhibits tumor progression and can increase therapeutic response by loosening the tumor extracellular matrix [50].

The direct cell killing effect of PDT, on both tumor parenchyma and stroma, has the potential to be impaired by the dependence of the generation of ROS on the presence of oxygen. With the unorganized growth of vasculature, not all tumor tissue is properly vascularised leading to insufficient delivery of both oxygen and PS. Similar tumor types had different PS distributions, depending on vascularity, which resulted in an increased PDT response in tumors with the most optimally distributed vascularity [51]. Impaired vascularity has proven to be an obstacle in direct PDT mediated tumor destruction. The tumor eradicating effect of PDT as seen in studies is probably also dependent on other mechanisms besides parenchyma and stroma destruction.

#### 2.3.2. Vasculature Effects

The formation of new blood vessels, or neovascularization/angiogenesis, is a key process in cancer development [52]. The importance of adequate tumor vasculature is evident with the existence of necrotic and low oxygen regions inside tumors due to the unorganized formation of blood vessels. Damaging existing vasculature or inhibiting the formation of new vessels has detrimental consequences for tumor proliferation, and anti-angiogenic therapeutics have been clinically approved for the treatment of cancers [53]. Damaging tumor vasculature has been shown to be an important factor in PDT efficacy. For example, much of the therapeutic effect of hematoporphyrin derivative (HPD) appears to be largely due to the consequences of disrupted blood flow [54].

Following PDT, endothelial and subendothelial cells are damaged. The direct damage to vasculature is significantly increased when the time between PS administration and light activation is shortened [55]. The influence of the interval between drug administration and light activation on vascular damage was investigated when the anti-tumor effect of verteporfin (Visudyne^®^) on rat chondrosarcomas was evaluated by varying the drug light interval (DLI). Long-term tumor regression was seen when verteporfin was activated 5 min after injection. Such long-term effects were not observed with activation 180 min after injection. This was ascribed to the acute vascular reaction seen at the 5 min interval due to PS activation within the blood vessels. Light treatment 180 min after verteprofin administration produced no apparent acute vascular response limiting the ischemic effect [55]. These studies clearly illustrate the impact of vascular damage on PDT induced tumor destruction.

Intravascular PDT damages endothelial cells, causing them to round up, widening the interendothelial cell junctions and exposing the underlying tissue. Damaged endothelial cells may release clotting factors such as von Willebrand factor, activating platelets [56,57]. The activated platelets interact with the exposed subendothelium leading to platelet aggregation, thrombus formation and vessel occlusion [54,58]. Moreover, activated platelets induce vasoconstriction after PDT, further decreasing blood flow [59]. The impaired blood flow and bloodvessel destruction, in time, will result in tissue hypoxia, nutrient deprivation and tumor destruction [54,55]. These features of PDT led some research groups to adopt the concept of vascular targeted PDT to increase therapeutic efficacy. In studies comparing cellular targeted PDT with a vasculature targeted approach by modulating the DLI, efficacy increased when tumor vasculature was targeted with a short DLI [55,60,61]. Increased efficacy was also seen when PDT and a vasculature targeted approach were executed in alternation to target both the tumor parenchyma and vasculature [61].

The enhanced therapeutic effect in these studies is probably due to longer lasting tissue hypoxia. When measuring the tissue oxygen levels during and after PDT and vasculature targeted PDT, pO_2_ drops significantly during both procedures due to the formation of ROS. However, in PDT, tissue oxygen levels quickly recover while after vasculature targeted PDT no such recovery is seen. Tissue reoxygenation after PDT lowers its therapeutic outcome which can be avoided by the destruction of blood vessels ensuring long-lasting hypoxia and a better therapeutic outcome [62]. The vasculature disrupting effects of PDT are an important component of PDT efficacy.

#### 2.3.3. Immune Reaction

The third mechanism of PDT-induced tumor destruction is the initiation of an inflammatory response that is followed by host tumor immunity. PDT-induced oxidative stress can upregulate the expression of heat shock proteins (HSPs), transcription factors related to inflammation and release of inflammatory cytokines [33]. Tumor cell death is accompanied by the release of proteins and other molecules, called damage-associated molecular patterns (DAMPs), that can elicit a strong inflammatory response. Studies show that after PDT, HSPs such as HSP70 are upregulated and are either expressed on the cell surface or in case of necrosis can be released extracellularly [63]. HSP can bind tumor antigens and interact with Toll like receptors (TLRs), which is a major route of activating antigen presenting cells (APC) [64]. In addition, these interactions regulate the expression of inflammatory and immune response genes [65]. The origin of HSPs and other DAMPs can vary depending on the subcellular location of PDT action [66]. Other DAMPs observed after PDT are membrane breakdown products such as lipid fragments and metabolites of arachidonic acid [67], ATP [68] or the ER protein calreticulin [69]. Innate immune cells such as macrophages, lymphocytes and dendritic cells (DCs) are recruited by DAMPs during inflammation, to remove cellular debris and promote tissue healing [70].

The increased expression and activation of transcription factors such as nuclear factor κB (NFκB) and activator protein 1 (AP1) is a key mechanism in inducing an inflammatory response following PDT induced oxidative stress. Multiple studies with different PSs showed upregulation of transcription factors after PDT, under which NFκB and AP1 (reviewed by [33]). These transcription factors induce expression of inflammatory cytokines such as tumor necrosis factor (TNF)-α, interleukin (IL)-6 and IL-1β that stimulate neutrophilia [71,72]. Moreover, an increase in the expression of cell adhesion molecules is seen that facilitates neutrophil migration [73]. The number of circulating neutrophils at the time of PDT plays an important role in determining PDT efficacy [74]. Not only do neutrophils remove photodamaged tumor cells, they also directly affect tumor-specific T cell proliferation/survival and mediate the generation of anti-tumor immunity following PDT [75].

Following the immediate inflammatory response of the innate immune system, host anti-tumor immunity can develop. Anti-tumor immunity was first discovered when lymph node cells from PDT treated mice were transplanted to naive hosts and induced suppression of subsequent tumor challenges. PDT-treated mice proved resistant against new tumor challenge after being tumor free for 100 days after PDT, indicating that an immune memory was established [76]. The importance of the lymphoid cells in long-term PDT efficacy was confirmed when all tumors were responsive to PDT and regressed in both immunocompetent and immunodeficient mice, but only regrew in the immunedeficient mice. Transfer of immunocompetent T-cells or bone marrow to immunodeficient mice resulted in restored long-term tumor sensitivity to PDT and delayed tumor recurrence [77]. Even though the adaptive immune reaction is not essentially important in the initial tumor damage, it primes the host for recurrences of similar tumors by formation of tumor-specific memory cells [78]. The acute inflammatory response following PDT, resulting in the phagocytosis of apoptotic and necrotic tumor cells, forms the basis for this mechanism. The immature DCs, that are part of the initial phagocyte army, remove cellular debris and dead cells, and are able to form and present antigens [79]. The interaction of DAMPs such as HSP70 with the TLRs on DCs can result in DC maturation and activation after which they are able to cross-present antigens [68]. As such, mature DCs are able to prime T-lymphocytes generating tumor-specific T-lymphocytes [80]. The antigen presenting DCs interact with CD4 helper T-lymphocytes that can subsequently activate CD8 cytotoxic T-lymphocytes (CTL), although T-helper cell independent activation of CD8 T-cells has been described [81]. The CTLs are able to recognize tumor cells and induce tumor cell death.

The ability to induce anti-tumor immunity after PDT has led to the search for PDT generated cancer vaccines. These vaccines could be used either prophylactic or even as therapy. Studies investigating the possibility of such vaccines have had some promising results [82,83,84]. Further experimental studies will prove the clinical applicability of this approach.

### 2.4. Photosensitizers

PSs have typically been divided in generations based on the time of development and their specific characteristics. The first generation PSs are the hematoporphyrins (Hp) that first arose in the 19th century. The first Hp, formed from dried blood, was a mixture of several porphyrins, each with their own characteristics. It was initially used as a fluorescent diagnostic tool for cancers but due to its heterotypical nature, large doses were needed to achieve desired effects. When processed further, a hematoporphyrin derivative was formed that had better tumor localization properties and could be used as a PS to treat gliomas by means of PDT. Another purification step resulted in the formation of porfimer sodium, or Photofrin^®^, which was approved by the U.S. Food and Drug Administration (FDA) and the European Medicine Agency (EMA) for use in the clinic to treat cancers and pre-cancers. Even though it is the most widely used PS for the treatment of cancers, Photofrin^®^ still is a complex mixture of molecules with relatively poor tissue selectivity, low absorption of light and poor tissue penetration of light. High Photofrin^®^ dosage is needed for therapeutic effect leading to prolonged patient skin sensitivity after PDT [85]. This led to the development of second generation PSs that were made to overcome the limitations of the first generation. They consist of all sorts of porphyrins generally divided into porphyrins, chlorins, pheophorbides, bacteriopheophorbides, texaphyrins and phthalocyanines, each group consisting of numerous types of PSs (reviewed by [86]). Second generation PSs aim to increase PS purity and reproducibility to have better control over production and drug behaviour. The goal of using these second generation PSs was to achieve better tumor selectivity and reduce the overall drug dose. The added effect of lower doses means the product is cleared faster and skin photosensitivity can be reduced from weeks to days. The photochemical properties of these new PS were adjusted so as to utilise the preferential absorption of light at longer wavelengths so they can be used to treat tumors in deeper tissues or utilize fewer implanted light sources [85]. A third generation PS refers to modified second generation PSs with biologic conjugates such as carriers, antibodies or liposomes to improve their physical, chemical and therapeutic properties. These compounds are often actively targeted towards the tumor resulting in higher selectivity. This will also ensure lower dosage and fewer unwanted side effects. PS have also been designed to absorb light of the best possible wavelength for ideal tissue penetration [87]. Although most sensitizers are porphyrins, either synthetics or derivatives, there is also a group of non-porphyrin PSs of which some are used in pre-clinical and clinical trials (reviewed by [86]).

### 2.5. What Affects PDT Efficacy

#### 2.5.1. Light

The therapeutic efficacy of PDT depends on the properties of the light used to activate the PS. In a superficial approach it has to both penetrate skin and tissue to reach the target site and be able to activate the PS in situ. In an intraluminal or interstitial setting the placement of multiple light sources is an important consideration. The penetration of light in tissue is a complex process, which is dependent on the optical properties of the tissue at the wavelength of light used. There is significant heterogeneity between tissues and even within tissues, with numerous molecules influencing light scattering and absorption. At shorter visible wavelengths, efficacy can be limited due to the absorption by endogenous chromophores such as haemoglobin, whereas at longer wavelengths water can absorb light. This limits the range of wavelengths to optimally penetrate tissue between 600 nm and 1300 nm. However, light with a wavelength longer than 850 nm doesn’t provide sufficient energy needed to activate the PS to its triplet state and to generate singlet oxygen. As such, the “therapeutic window” for the majority of PDT applications lies in the red region of the spectrum between 620 and 850 nm achieving optimal tissue penetration and PS activation [88].

For the delivery of light, both lasers and incandescent light have proven to be effective [89]. It is improbable that a single light source could cover all types of PDT and the source used should be fitted to the PS photophysical characteristics (absorption spectrum), type of disease (location, size of tumor, tissue type) and usability (cost, size, handling). With topical lesions, at for instance the skin or oral cavity, it is easier to use a lamp instead of a laser since they are cheaper to maintain, user friendly and their broad emission can be used with several PSs [89]. Lasers are widely used in clinical PDT as they are powerful, can be coupled to optical fibres that can be used to interstitially illuminate deeper located tumors with the application of diffusing tips [89]. Numerous studies are looking to optimize light sources with new approaches. For instance, the use of Light Emitting Diodes (LED) in PDT is investigated [90,91,92]. LEDs are cheap, easy to manufacture, have a high power output and can be used for a broad range of wavelengths. They are however of limited use for large tumors where an interstitial approach is required. Recently, the use of daylight for topically applied PDT is an active field of investigation [93,94]. Because the spectral intensity of daylight contains a large proportion of blue light, which does not penetrate significantly, it remains to be seen if this is an applicable approach beyond its use with porphyrin pre-cursors and superficial skin (pre-) malignancies (described later in this review).

Besides the light source itself, the manner of application is important. Different irradiation protocols with the same light source can have different outcomes in PDT. High fluence rates can deplete the oxygen levels in tumor tissue too fast, limiting the volume of tumor reached with PDT [95,96]. Light fractionation, for example with the use of porphyrin pre-cursors has been the subject of significant investigation [97]. Moreover, light dose regimens might also influence the host anti-tumor reaction [98]. Optimal dose regimens are likely case dependent. Therefore, a full understanding of light dosimetry is an important part of PDT. The subject of light in PDT is under careful investigation and improvements and new technologies in this field will improve overall PDT efficacy [99,100] and protocols [101,102].

#### 2.5.2. Oxygen

The availability of sufficient tissue oxygen is crucial in the efficacy of cancer therapy. The presence of hypoxic areas in tumors proves to be a major obstacle in the treatment of solid tumors [103]. One indirect reason is that hypoxia is usually induced by impaired tumor vasculature, meaning drug delivery routes are impaired. Another reason is the importance of oxygen for the therapeutic effect of for instance radiotherapy and certain chemotherapies [103,104]. In PDT, the formation of singlet oxygen needs ground state oxygen, therefore tissue oxygenation heavily influences PDT efficacy [105,106]. Some PSs precursors such as aminolevulinic acid (ALA), are more effectively metabolized to the active PS, protoporphyrin IX (PpIX), in oxygen rich environments [107]. Consequently, hypoxic areas inside tumors have proven obstacles for PDT efficacy and tumors with hypoxic areas are considered PDT resistant [108]. Indeed, when the main vasculature of a tumor is occluded, the effect of PDT is considerably ablated [109]. Increasing tumor oxygenation by hyperbaric oxygen therapy has been shown to improve tumor response to chemotherapy and radiotherapy [110]. In PDT studies there are conflicting reports. When Photofrin^®^ is used in combination with hyperoxygenation by letting tumor bearing mice breathe under pressurized conditions, improved cell killing after PDT is observed [111,112]. Other PSs show the same oxygen dependent efficacy [113] while yet others seem to be unaffected by lower tissue oxygen levels [114]. This suggests that changing the partial pressure of oxygen in the blood has little effect on the oxygenation of cells distant from the microvasculature where oxygen is needed for PDT.

The formation of ROS results in oxygen depletion during PDT depending on light fluence rate [95,96]. Furthermore, the vascular collapse following PDT adds to the decrease of tumor oxygenation [54,115]. By adjusting the light and PS dosimetry, issues relating to these occurrences might be circumvented [96].

#### 2.5.3. PS Uptake and Localization

With the limited action radius of ROS and especially singlet oxygen, the precise localization of the PS can be crucial for its therapeutic effect. Understanding and controlling PS localization greatly increases the potential of PDT. From the moment of administration, until the PS has reached the target location, various physical, chemical and biological events take place that together influence the end location of the PS. For example, when intravenously administered, the PS will first encounter serum proteins to which it will bind. Different PS will associate differently to these proteins and therefore the pharmacokinetics and distribution will vary accordingly [44]. The PS has to extravasate the blood vessels to reach the tumor site, thereafter associating with the extracellular matrix or the cells within the tumor. As mentioned earlier, PSs have been found to localize in numerous organelles which is dependent on the structural characteristics of the PS. It has been shown that overall charge, charge distribution, lipophilicity and overall structure predominantly determine cellular uptake and subcellular localization of a PS and ultimately determine its therapeutic effect [116].

##### Charge

The net charge of a PS determines the interaction between PS and cellular membranes. As cellular membranes are negatively charged, negatively charged PSs have decreased transmembrane transport compared to positively charged PSs, which readily cross membranes [117]. Cationic PSs diffuse freely across the plasma membrane and, in the cell, predominantly localize towards the membranes of mitochondria [118,119]. Porphyrins of different charge and charge distribution were compared for their uptake and localization and the mono-cationic porphyrin localized towards membranous compartment of the plasma membrane, Golgi, mitochondria and ER, while the more positively charged preferentially localized in the mitochondria [120]. Instead of passive transport followed by mitochondrial localization, anionic PSs are taken up via endocytosis, which leads to localization towards lysosomes [118]. Low negative charges can be compensated by lipophilicity [21].

The importance of charge distribution becomes clear when molecules of similar charge but different distributions are compared. A different location of a charge on the molecule might interfere with its availability and impair the electrostatic interaction between PS and membranes. Changing the location of side chains on Zn(II) meso-tetrakis(N-alkylpyridinium-2(or -3 or -4)-yl)porphyrins altered their interactions with cellular membranes and thus their uptake and distribution in the cell [121]. Altering PS overall charge and charge distribution appears to be useful in determining its subcellular localization and therefore photokilling efficacy.

##### Lipophilicity

Studies show that altering the lipophilicity of a PS affects its plasma distribution and, consequently, affects its uptake and localization. The more hydrophilic photosensitizers mostly bind albumin, whereas the amphiphilic PS bind high-density lipoproteins, and the hydrophobic ones, that are administered with a solubilisation vehicle, mostly localize in the inner lipid core of low-density lipoproteins [44]. At the tissue level, increased lipophilicity generally contributes to higher uptake. For instance, lengthening the side chains of Zn N-alkylpyridylporphyrins increased their lipophilicity 50 times, resulting in increased uptake and efficacy [121]. Lipophilicity also influenced subcellular localization as it moved from predominantly localizing to lysosomes towards mitochondria with increasing amphiphilicity. The increased preference for membrane interactions of more lipophilic compounds increases their localization towards mitochondria [122].

##### Three Dimensional Shape

PS uptake is also dependent on the tri-dimensional shape of the molecule as different analogues with similar charge and lipophilicity showed different characteristics. This was probably due to the spatial availability of charges [121]. While investigating the uptake of PS homologues with variable lipophilicity, it appeared that homologues with similar lipophilicity but of differing structures are taken up in different manner [123]. Moreover, some molecules with higher lipophilicity were taken up less than less lipophilic homologues, indicating other characteristics are important for cellular uptake. Therefore, besides charge and lipophilicity, the structure of a molecule may play an important role in tumor uptake and PDT efficacy.

### 2.6. An Ideal PS

The definition of an ideal PS is often described based on preferential characteristics. Several properties are generally accepted as ideal [21,124]. The PS should have low dark toxicity and preferably no toxicity at administration (no allergic reactions or hyposensitivity). Moreover, administration should be easy and feasible via different routes without any pain. It should have a high absorption band, preferably in the near infrared (NIR), for optimal tissue penetration, yet with enough energy to generate singlet oxygen. It should have a high yield of ROS during illumination. High tumor selectivity and rapid clearance from the body will minimize photosensitivity of the skin. Moreover, the PS should be pure and easily produced, as well as be stable enough for long storage. The search for new and improved PSs is an active field of research as can be seen by the currently ongoing clinical trials looking to assess safety and efficacy of newer PSs and the many preclinical reports of completely novel PSs. One of the focus points is water solubility to improve PS circulation and efficacy in aqueous surroundings. By rationally designing molecules it is possible to synthesize water soluble PSs that also accumulate at desired locations [125]. Adding functional groups to the PS allow bio-conjugation of moieties capable of accentuating desirable properties [126]. Rather than specific designs, library screening might lead to interesting new compounds that have favorable characteristics compared to currently used PSs [127,128].

## 3. PDT in Clinical Trials

### 3.1. Clinically Approved PS

In the clinic, PDT can be used in conjunction with surgery, radiotherapy (RT) or chemotherapy (CT), due to its mode of action. Because it is activated locally and has limited tissue penetration, PDT is relatively tissue sparing with, in some cases, good cosmetic outcomes. This makes it especially suitable for skin conditions and sensitive areas such as the head and neck [129]. Moreover, it lacks the adverse events (AE) seen in RT and systemic CT. Unfortunately, intravenously administered PSs induce prolonged periods of skin photosensitivity, during which patients need to avoid light [129].

Despite PDT having several favourable characteristics to standard treatment modalities, only four PSs have received regulatory approval for the treatment of cancers by the FDA and EMA. Porfimer sodium (Photofrin^®^) was the first PS to get clinical approval for the treatment of several indications (Table 1). However, Photofrin^®^ is a complex mixture of molecules with relatively poor tissue selectivity, low absorption of light and, at the wavelength needed for Photofrin^®^ activation, light has poor tissue penetration. A high dosage of Photofrin^®^ is needed to achieve the desired therapeutic effect, leading to long circulation times and prolonged patient photosensitivity [21]. The second PS to receive approval is the second generation temoporfin (Foscan^®^ or mTHPC), which has been approved by the EMA for the treatment of advanced head and neck squamous cell carcinomas. It absorbs light at longer wavelengths and has shorter circulation time, improving its safety profile compared to first generation Photofrin^®^.

Several formulations of aminolaevulinic acid (ALA) have been approved for dermatological indications. 5-ALA is licensed for the treatment of actinic keratoses while its methyl-ester derivative methyl-ALA (MAL) is used to treat non-hyperkeratotic actinic keratoses, Bowen’s disease, and superficial and nodular basal cell carcinomas. Due to their local or topical application, ALA derivatives have a favorable safety profile compared to Photofrin^®^. However, these are only indicated for superficial lesions due to their limited tissue penetration. There are a few other PSs approved for indications beyond the scope of this review, as summarized in Table 1.

### 3.2. Organ Specific PDT in Clinical Trials

Even though PDT has been investigated for decades, only few PSs are approved for use in a clinical setting, as described earlier. However, recognizing the potential of PDT, investigators are trying to evaluate the safety, feasibility and efficacy of a variety of PSs for PDT for numerous types of cancer and increase utilization of PDT in the clinic. Ongoing clinical trials, as registered on www.clinicaltrials.gov, focus on the safety and protocol optimization of PSs that have been under investigation for many years. Using the search-terms “photodynamic therapy” and “cancer” and selecting for open trials, 58 ongoing trials can be found using 11 different PSs in different types of cancer. Most trials are trying to establish the optimal dosage of PS or administered light for compounds clinically approved for either other types of cancer or in other parts of the world, with Photofrin^®^ and ALA derivatives being used in the majority of the studies. The next section will discuss trials and studies done during the last ten years that investigate the use of PDT for cancer in a clinical setting. Where relevant, the currently ongoing clinical trials are also described to illustrate the direction of PDT research.

Supplementary Table S1 provides an overview of our review findings, summarizing tumor type, study goal, methodology, PS, outcome, and adverse events for clinical studies in each organ.

#### 3.2.1. Lung

Cancers of the lung are one of the leading causes of cancer-related deaths worldwide with nearly 1.8 million diagnoses and 1.59 million deaths in 2012 [130]. Surgery is the first choice treatment, and is for the majority of tumor types, the only curative intervention for early diagnosed lung cancer. However, patients are usually diagnosed with late stage, unresectable disease where lung function sparing, palliative treatments such as CT and RT are preferred [131]. In case of inoperable disease and failure or refusal of other treatments, PDT has potential as a palliative standalone or combination therapy due to lack of systemic effects and its organ-function sparing action. Additionally, as opposed to RT, the working mechanism of PDT allows repeated treatments.

PDT was deemed well-tolerated and effective as part of a multi-modal treatment for endobronchial non-small cell lung cancer (NSCLC) in a small retrospective study [132]. Photofrin^®^-PDT combined with high dose rate brachytherapy (HDR) achieved prolonged local tumor control. With the right protocol (concerning order and dosage of interventions), PDT following HDR can achieve tumor control for longer periods of time compared to other modalities or either treatment alone [132].

The palliative efficacy and safety of PDT as part of a multi-modal treatment was evaluated in a single centre prospective pilot study with patients suffering from advanced NSCLC with central airway obstruction [133]. PDT consisted of an intravenously administered water-soluble chlorin E6 complex (Radachlorin^®^; Rada-Pharma, Moscow, Russia) followed by endoluminal irradiation via fibroptic bronchoscopy. All patients showed improvement of their symptoms with significantly improved lung capacity and function. One year post-PDT, survival was improved significantly for PDT treated patients compared to the one-year survival rate mentioned for patients with NSCLC treated with systemic CT alone [133,134].

Talaporfin-PDT, which received approval as a lung cancer treatment in Japan, was combined with chemo-radiation therapy (CRT), RT or CT to palliatively treat intractable lung cancer with airway stenosis. This multimodal approach significantly relieved airway obstruction, improved lung capacity parameters and quality of life (QoL), ultimately prolonging patient survival [135].

Neo-adjuvant therapy is often given in an attempt to shrink tumors and improve the chance of successful surgery. The addition of Radachlorin^®^-PDT to preoperative CT significantly increased the number of patients eligible for radical resection compared to neoadjuvant CT alone [136]. PDT could also be a valuable addition to the adjuvant therapy regimens. A study showed patients receiving postoperative Photofrin^®^-PDT have an improved mean survival time when compared to patients treated with standard postoperative care [137]. In a different study, mesothelioma patients undergoing radical pleurectomy followed by post-operative PDT showed unusually long survival, despite recurrences and no increased progression-free survival, most likely due to the preservation of the lung and/or the PDT effect [138]. These studies indicate PDT can be easily implemented in standard care regimens, either pre- or postoperative, to improve therapy outcome.

As a stand-alone treatment, Photofrin^®^-PDT proved a good alternative for palliative CT or RT in unresectable lung cancer as it achieved an overall response of nearly 87% and improved patient QoL. It even showed curative potential with several cases of complete remission (CR) [139]. However, photosensitivity, secretions and pain were common adverse effects (AEs). Another major drawback of Photofrin^®^-PDT was the fact it becomes less effective with tumors over 1 cm in diameter [140,141]. As such, the guidelines of the American College of Chest Physicians in 2003 recommended that PDT is only suitable for lesions under 1 cm in diameter based on results with Photofrin^®^. However, second generation PSs with deeper tissue penetration prove more effective with larger lesions. No significant difference in efficacy was observed between tumors under or over 1 cm when using talaporfin [142]. The same group showed that talaporfin-PDT was also effective in treating patients with multiple primary lung cancer (MPLC). All MPLC patients that received PDT, either alone or in combination with surgery, achieved CR indicating PDT can be used for multiple lesions under certain conditions [143].

Other, newer PSs such as 2-[1-hexyloxyethyl]-2-devinyl pyropheophorbide-a (HPPH) are also finding their way to clinical trials. HPPH is a chlorin based PS which absorbs light at 665 nm and has a lower risk of skin sensitivity due to its shorter half-life compared to Photofrin^®^ [144]. A Phase I dose escalation study showed HPPH-PDT is capable of achieving high rates of CR that is retained for months in patients with carcinoma in situ (CIS) and micro invasive cancer (MIC) of the central airways [145]. Minor photosensitivity was reported but overall AEs were limited.

Two ongoing clinical trials are investigating two new PSs for their safety and efficacy in lung cancer. One trial is investigating the water-soluble palladium-bacteriochlorophyll WST11 in obstructive NSCLC (EudraCT ID: 2009-011895-31). WST11 should have improved efficacy compared to older PSs and fewer side effects due to rapid clearance [146]. The other study is an open-label Phase IIb study to evaluate the safety, tolerability and efficacy of Fotolon^®^ (Chlorin e6-PVP) for the treatment of obstructing NSCLC (EudraCT ID: 2013-001876-39).

Recently a clinical trial has been launched to evaluate the safety and feasibility of using navigational bronchoscopy to perform interstitial PDT using Photofrin^®^ as treatment in patients with unresectable Stage IA peripheral non-small cell lung cancer (NSCLC, Clinicaltrials.gov ID: NCT02916745).

Overall, Photofrin^®^-PDT proves effective as a palliative treatment in lung cancer, yet is associated with prolonged skin photosensitivity. Moreover, it proves less effective with larger lesions. In contrast, newer PSs like talaporfin and HPPH that have higher absorption bands at longer wavelengths, show increased efficacy making them suitable for cases where first generation Photofrin^®^ fails. An added advantage of these second generation PSs is the decreased half-life leading to shorter photosensitivity periods and fewer cases of related AEs.

#### 3.2.2. Esophagus

Oesophageal cancer accounted for 3.2% of the newly diagnosed cancers in 2012. With a very poor mortality to incidence ratio, it is the sixth most common cause of cancer related death (4.9% of total) [130]. Esophagal cancer histology differs by location: esophagal squamous cell carcinoma (ESCC) is located in the upper and middle part of the esophagus while adenocarcinoma (ADC) is mostly located in the lower part. Worldwide, ESCC is the most prevalent form of esophagal cancer but with the increasing prevalence of obesity and the associated gastro-esophageal reflux disease (GERD), an increase of ADC is seen in western countries [147,148]. GERD increases the chance of developing Barretts esophagus (BE), an early precursor for ADC. Locally advanced esophageal cancer can be surgically removed by esophagectomy in operable patients but postoperative morbidity and mortality occur regularly and long-term outcome is poor [149]. Only small advantages of neoadjuvant CT and CRT to improve treatment outcome have been observed [149,150]. Peri-operative CT or CRT appears beneficial but the advantage was more pronounced for younger patients as no survival advantage was seen for the elderly patients [151]. CRT is used as definitive treatment option for ESCC but residual or recurrent lesions remain a major obstacle showing improved therapies are still needed. Moreover, reducing morbidity associated with CT would also improve current treatment strategies, which is why PDT has great potential.

Several clinical studies show the curative potential of Photofrin^®^-PDT for BE and early esophagal cancer. In a retrospective study, Photofrin^®^-PDT applied with curative intend proved successful in BE patients with high grade dysplasia (HGD), an indication with a higher chance of progression to cancer. It was less effective in patients who had ADC or ESCC, especially with larger lesions [152]. A similar study supports this data by stating PDT proved effective in treating smaller BE or ADC lesions but complete ablation was less likely with lesions over 3 cm in length [153]. Maybe even more important than BE length is esophageal wall thickness, as thicker walls have a lower chance of achieving successful results with Photofrin^®^-PDT [154].

Histology is currently the general way to predict treatment efficacy and assess treatment response. It is attempted to find other predictors to be able to improve patient selection and treatment outcome prediction. Some genetic biomarkers could possibly be used to predict PDT efficacy as a loss of allelic p16, which encodes for a protein involved in apoptosis regulation, is correlated with a decreased response to PDT [155]. In line with these results, it was found that the prevalence of certain biomarkers after successful PDT could predict the chance of recurrence. Amplification of proto-oncogene loci was associated with an increased chance of HGD recurrence after an initial histological response to PDT [156]. Photofrin^®^-PDT is indeed effective but it appears that taking lesion length, thickness and possibly several genetic biomarker levels into account when establishing PDT dosage, can still improve Photofrin^®^-PDT efficacy. It is considered an effective component of adjuvant therapy for patients with dysphagia that are unfit for, or refuse, surgery. As part of a multimodal approach, Photofrin^®-^PDT has shown good results with improving the patients’ QoL by effectively and immediately palliating dysphagia [157].

In case of failure at the primary tumor site after CRT or RT, Photofrin^®^-PDT has also shown potential as a salvage treatment [158,159]. It is considered a better alternative for salvage esophagectomy, as surgery has higher morbidity and mortality rates as a salvage treatment compared to when it is a primary or planned intervention [160]. Moreover, a retrospective study investigating long-term survival of patients receiving either surgery or PDT stated that the overall survival was comparable between the interventions [161]. Indeed, BE patients with HGD that are unfit or otherwise not suited for surgery or other modalities can achieve long-term tumor free survival after Photofrin^®^-PDT [162]. However, post treatment morbidities associated with Photofrin^®^, such as stricture formation and photosensitivity, result in the preference for other modalities such as radiofrequency ablation (RFA) [162]. Other therapies such as endoscopic mucosal resection (EMR) and RFA show similar efficacy and better safety compared to Photofrin^®^-PDT and became increasingly popular [160]. A large cohort retrospective study showed that Photofrin^®^-PDT was more effective in achieving a complete response in HGD and ADC patients compared to other modalities. However, the morbidities related to Photofrin^®^ and the additional financial investment needed for PDT, drive clinicians to use other modalities such as RFA and EMR [163]. Recognizing the potential of PDT, it remains an active topic of research. Not only do better protocols help with increasing Photofrin^®^-PDT efficacy and safety, the rise of second generation PSs renewed the interest in using PDT for esophagal cancers [160].

A randomised controlled, dose-finding study showed comparable or even better efficacy using 5-ALA compared to Photofrin^®^ in patients with HGD. Moreover, PDT using 5-ALA was carried out without the complications seen with Photofrin^®^ due to improved localisation [164]. A follow-up study also showed comparable efficacy of 5-ALA to Photofrin^®^ for HGD in BE, but RFA was still considered superior [165]. This study is still ongoing (EudraCT ID: 2005-005528-15).

Other, newer PSs also show improved safety profiles compared to Photofrin^®^ and are often associated with better efficacy. In a dose finding study, HPPH showed reduced photosensitivity combined with increased efficacy compared to Photofrin^®^. After one round of HPPH, all patients with precancerous lesions and early intramucosal cancer associated with BE, achieved CR initially and after 5 years, 39% was still tumor free [166]. In a Phase I study, talaporfin-PDT proved to be safe and effective as a curative treatment option for ESCC patients with local failure after definitive CRT [167]. Compared to Photofrin^®^, talaporfin achieved similar efficacy with considerably less morbidities, but proved less effective for larger lesions. However, with Photofrin^®^-PDT, this decreased efficacy with bigger lesions is more pronounced [168].

In summary, Photofrin^®^-PDT is approved with curative intent for BE, as palliative treatment for advanced obstructive esophagal cancer or as salvage treatment after failure of other modalities. The use of Photofrin^®^-PDT for precancerous lesions and (early) esophagal cancer is effective, however, it is often accompanied by patient photosensitivity and reduced efficacy with larger lesions (see Supplementary Table S1). Studies with second generation PSs show better efficacy and lower morbidity but additional trials are needed to potentially implement them as first-line treatment of esophagal cancer.

#### 3.2.3. Skin

Skin cancers can be divided over two groups called melanomas or nonmelanoma skin cancers (NMSC). The primary cause of skin cancers is, in more than 90% of the cases, exposure to ultraviolet radiation from the sun [169]. Malignant melanoma arise from pigment-containing cells and about 25% develop from moles [170]. Treatment of the highly aggressive melanomas is predominantly wide surgical excision or, in case of stage III or higher, radiation or chemotherapy. NMSC are, in general, less aggressive and can be further separated in two subgroups based on their origin, basal cell and squamous cell skin cancers. Current treatment options for NMSC include surgical excision (considered the gold standard), curettage and electrodessication, cryosurgery, radiotherapy, topical chemotherapy (5-fluorouracil) and immune modulating agents (Imiquimod^®^), photodynamic therapy (PDT) and Mohs’ micrographic surgery. The choice of treatment for NMSC is determined by factors like the number and size of lesions, location, and patients’ preferences with respect to treatment options. PDT with its good cosmetic outcome, repeatability and high response rate may be a good alternative to the golden standard surgery.

The use of PDT for the treatment of skin cancer has a long history. Dougherty et al. already described in 1978 the use of HPD PDT for the treatment of skin and other cancers [12]. The promising results, good cosmetic outcome and high repeatability of PDT combined with the easy accessibility of skin to treatment and observation made the development of PDT for skin cancer an ideal research field. Photofrin II (PII), a purified preparation of HPD, mediated PDT has shown complete responses up to 85% for nodular and superficial Basal Cell Carcinoma’s (BCC) but the prolonged skin photosensitivity is a serious adverse effect [171]. In late 80th and early 90th of the 20th century Kennedy et al. started researching the use of the porphyrin precursor 5-aminolevulinic acid (ALA) for PDT [172]. The use of this second generation photosensitizer results in good clinical and cosmetic outcome without the adverse effect of the prolonged skin-photosensitivity seen after HPD or PII. In a review, Zeitouni et al. reported that ALA-PDT leads to an average of 85% response rate in nodular and superficial BCC’s from papers published between 1978–1993 [171]. While initially the short term responses (<1 year follow-up) were excellent, the long term responses showed considerable room for improvement. A range of approaches were explored, predominantly pre-clinically, to enhance the treatment response either by increasing the accumulation of protoporphyrin IX (PpIX) by the use of a pretreatment, for example, iron chelation, tapestripping, DMSO, ionthophoresis [173] or esterified ALA [74] or a nanoemulsion formulation [174]. Of the esterified ALA’s methyl aminolaevulinate (MAL) is the most commonly used for treating skin cancers. A different route to improve the response to ALA-PDT is to illuminate according to a light fractionation scheme [175].

Various clinical studies are performed investigating PDT for the treatment of BCC’s comparing it to other treatment options. ALA shows comparable effectiveness to MAL-PDT of NMSC’s when determined at a relative short follow up of 8 weeks or 6 months respectively [176,177]. Surgery, being the golden standard, showed no inferiority over MAL-PDT in the complete response rate 3 months after treatment of sBCC in a group of 196 patients [178]. The cosmetic outcome was however significantly better after MAL-PDT. In a different study, MAL-PDT showed to be less effective than Imiquimod^®^ crème with a follow-up of 12 months without loss of cosmetic outcome and despite the fact that patients compliance was less with Imiquimod^®^ [179]. The five year response of sBCC to a single round of ALA-PDT treatment was significantly improved by applying a light fractionation scheme (88.4% CR after light fractionation vs. 75.4% CR after a single illumination) [180].

The only side effect of ALA-PDT reported is a burning, itchy sensation during illumination that in rare cases is so severe that patients discontinue the treatment. Reports comparing pain after MAL and ALA are sometimes in contradiction. No difference in pain was observed in patients treated for nBCC with or without debulking the tumors [176]. A reduction in pain during illumination of sBCC’s could be accomplished by delivering the light according to a two-step irradiance schedule for both ALA and MAL [181,182]. Illumination is started with a low fluence rate until 90% of the fluorescence is photobleached after which the fluence rate is increased until the total fluence is delivered.

Actinic keratosis (AK), also known as solar keratosis, is a scaly lesion typically located on sun-exposed areas that can, if left untreated, develop into squamous cell carcinomas in 10%–20% of the cases. This risk factor has lead Dermatologists to investigate the use of topical chemotherapy, cryotherapy or photodynamic therapy. ALA-PDT has also been investigated for the treatment of AK’s on the scalp or fore head. ALA shows comparable effectiveness to MAL-PDT as determined at 1 month follow-up [183]. However significantly more pain was observed after ALA compared to MAL [183,184]. BF-200 ALA, a nanoemulsion based gel formulation containing ALA, has shown to be significantly better or comparative with MAL in the treatment of AK [185,186]. However here also BF-200 ALA (Ameluz^®^) resulted in significant more pain than MAL [187]. Recently the use of daylight for the illumination of lesions is investigated and compared between MAL and BF-200 ALA and found to be equally effective or better for BF-200 ALA with no difference in pain experience [188]. The safety and efficacy of BF-200 ALA compared to HAL and MAL for superficial BCCs and AK is currently under investigation (ClinicalTrials.gov: NCT02367547 and NCT02647151). Another ongoing clinical trial is focusing on optimizing MAL PDT for basal cell carcinoma for complete cancer cell eradication (EudraCT ID: 2016-002508-16).

Overall PDT using PpIX precursors like ALA, BF-200 ALA or MAL to treat specific NMSC skin cancers results in good clinical and excellent cosmetic outcome and represents an attractive alternative to surgery.

#### 3.2.4. Head and Neck

Head and neck cancers (HNC) are a heterogeneous group of cancers with tumors in the oral cavity, pharynx, larynx, nasal and sinus cavities, orbit, and other related structures like the skin. They commonly arise in the mucosal linings and may impair the patient’s ability to breathe, swallow, drink or eat. Treatment of HNC is therefore not only dependent on type, but also location and stage of the disease. The complexity of the head and neck region with its various critical structures and complex architecture puts limitations to the treatment execution. Standard care consists of either surgery, RT or systemic therapy like CT, CRT or molecular targeted agents [189]. The survival rate, however, after surgery and radiotherapy is moderate [190]. Surgery is invasive and is sometimes associated with severe morbidity, while CT and RT are aggressive and due to limited selectivity have side effects reducing patients’ QoL. As such, PDT is an interesting alternative being less invasive with good cosmetic effect and with lower morbidity [191].

Initially PDT was explored for treating early stage, easy to reach, head and neck cancer and focused on superficially growing basal cell carcinoma (BCC) and squamous cell carcinoma (SCC) using HPD [12,192,193]. Photofrin^®^ has been shown to be effective in the treatment of oral SCC and dysplasia but post-PDT photosensitivity and limited tissue penetration confirm the preference for second generation PSs [194]. The use of second generation photosensitizers like 5-aminolevulinic acid and mTHPC (Foscan^®^) for early stage HNC results in good clinical and cosmetic outcome without the adverse effect of the prolonged skin-photosensitivity seen after HPD and PII [195,196]. With HPPH, a different second generation photosensitizer, PDT has also been used for high risk dysplasias, CIS and oral squamous cell carcinoma (OSCC) and showed promising CR rates and mild AE [197]. Recently a Phase Ib study showed that HPPH-PDT can be safely used to treat early stage larynx cancer with 85% CR at the maximum tolerated dose [198]. Secondary to that the use of mTHPC with its far-red absorption combined with the possibility to illuminate interstitially meant that also thicker lesions could be treated [199,200].

Precancerous and low risk oral lesions have cure rates comparable to those obtained with primary surgery. Using 5-ALA (on thin to moderate dysplasia lesions) or mTHPC (on moderate dysplasia to severe dysplasia to CIS), PDT effectively resulted in CR or disease stabilization for prolonged periods of time [201]. For low risk lesions, long-term CR can be achieved by mTHPC-PDT with three and five year survival rates of 92.1% and 84.2% respectively, comparable to standard care [202]. In recurrent respiratory papillomatosis patients that need surgery every 3 months, mTHPC-PDT resulted in marked improvement of laryngeal disease across time with recurrences after 3 to 5 years [203].

Several studies were performed investigating mTHPC-PDT for the treatment of OSCC and oropharynx squamous cell carcinoma. Complete response rates of 96% and 86% have been reported for carcinoma of the lip and oral/oropharynx SCC after a follow-up of 12 months and 37 months respectively [196,204]. Both studies report good functional and cosmetic outcome. In a separate study it was shown that OSCC accompanied by field change disease responds less than single lesions and in some of the cases persistent disease remained [205]. Two studies have compared surgery with mTHPC-PDT for oral SCC on the tumor response, disease free survival and local or overall survival. OSCC ≤ 5 mm thick show no difference between surgery and mTHPC-PDT [206]. However, in a different study differences are reported on the need for re-treatment and local disease free survival. Still, PDT and surgery showed similar overall survival rates [207].

Recurrent and persistent nasopharyngeal carcinoma (NPC) and advanced HNC are a serious problem in HNCs as conventional treatment options are often exhausted. With the development of different illumination strategies like the nasopharynx applicator but also interstitial illuminations these lesions were accessible to PDT treatments. Treatment of nasopharyngeal carcinoma with mTHPC-PDT can result in CR and lead to prolonged survival for patients [208]. A prospective study using ultrasound guided interstitial PDT for deep seated pathologies showed good results with most patients reporting improved QoL and improvement of limb function [209]. In another study, interstitial PDT in a group of 45 patients with persistent or recurrent HNC prolonged survival of the 33 patients (73%) who responded to the treatment to a median of 16 months [200]. mTHPC is approved in Europe for the palliative treatment of advanced HNC, often when surgery or RT are no longer viable options. It proved effective in inducing tumor reduction and CR was achieved in half of the patients with HNC that had exhausted all other curative treatment options. Moreover, PDT stabilized disease leading to a significantly prolonged survival in patients that responded to treatment compared to non-responders confirming mTHPC-PDT can be considered as a valuable treatment option [210]. A recent report describes a small but impressive study investigating the use of mTHPC-PDT for the treatment of recurrent SCC of the base of tongue after salvage surgery following (chemo-) radiation failure. The two patients treated were disease free after 24 and 42 months follow-up [211]. mTHPC-PDT has also been shown to be a safe adjuvant therapy for recurrent malignant tumors of the paranasal sinuses and recurrent sino-nasal skull base tumors [212,213].

Overall, mTHPC-PDT shows efficacy that is comparable to surgery for early stage HNC and smaller lesions. For larger lesions, surgery is more effective on the long-term but is accompanied by high morbidity. As a palliative treatment PDT is preferred over standard care. Further studies with new and improved PSs and PDT protocols might enhance PDT efficacy in later stage HNC. In the search for other new and possibly improved PSs for PDT of HNC, a clinical trial is ongoing with Redaporfin^®^ (LUZ11), which will investigate the tolerability, pharmacokinetics and anti-tumor effect in a dose escalation study (EudraCT ID: 2013-003133-14).

#### 3.2.5. Bile Duct

Bile duct cancer (BDC) or cholangiocarcinoma (CC) is a rare form of cancer that can either be classified as extrahepatic (ECC) or intrahepatic (ICC) based on its anatomic location. Both ICC and ECC are difficult to diagnose as ECC is usually only symptomatic in advanced disease state, while ICC is mostly asymptomatic per se [214]. Resection is the recommended curative treatment but because the disease is usually diagnosed in advanced state, only 10%–20% of diagnosed patients are surgical candidates [215]. In patients with unresectable CC, palliative biliary decompression (by catheter or stenting) combined with RFA, CRT or PDT may improve survival and QoL [214].

Studies using PDT for treating CC started over two decades ago. A case report published in 1991 describes the first successful application of PDT for a patient with bile duct cancer where dihematoporphyrin ether was administered over the course of 4 years [216]. A meta-analysis study reviewing the reports comparing biliary stenting with PDT vs. biliary stenting only for CC, concluded that the combination resulted in significantly longer survival, higher QoL, and lower bilirubin levels [217]. In a study with patients with unresectable lesions, talaporfin-PDT appeared safe and effective when combined with standard care in treating BDC and improved survival of the patients. It also showed a better safety profile than Photofrin^®^-PDT [218]. Although it is not officially indicated as such, Photofrin^®^-PDT is considered standard care for unresectable BDC. However, due to its limited tissue penetration, Photofrin^®^ is not effective in destroying deep seated tumor cells [219]. The potential of Photofrin^®^-PDT for CC is currently under further investigation in a randomized Phase III clinical trial comparing Photofrin^®^-PDT followed by stenting with stenting + CT (ClinicalTrials.gov ID: NCT02082522).

In the first stage of a Phase II trial, the use of temoporfin (Foscan^®^)-PDT showed increased tumoricidal effect due to deeper tissue penetration, resulting in a better tumor response, prolonging the time to local tumor progression and increasing patient survival times [220]. The deeper tissue penetration of Foscan^®^ can, however, lead to perforation of hollow organs such as the bile duct. Therefore, it would be wise to recommend an adjusted dosage for BDC. During the second stage of this Phase II study, safety and long term efficacy of temoporfin compared to porfimer were evaluated in larger patient groups. The main findings of this study include the safe delivery of temoporfin in patients, resulting in less adverse effects, prolonged patency of hilar bile ducts, reduced number of PDT procedures and extended time to local tumor progression, compared to porfimer-PDT [221]. In addition, low dose-Foscan^®^ proved comparably effective to Photofrin^®^ in prolonging patient OS and improving QoL in unresectable BDC, but without any of the Photofrin^®^ associated side effects [222].

Addition of CT to Photofrin^®^-PDT for palliative treatment of unresectable BDC seems to have clinical benefits. The combination significantly improved 1-year survival rate, prolonged overall survival and prolonged local disease free survival compared to Photofrin^®^-PDT alone [223]. This was also seen in a recent retrospective study where patient survival times were significantly higher following PDT+CT compared to PDT alone [224].

Endoscopy-guided PDT using porfimer or talaporfin sodium was safely and definitively performed in 25 patients with bile duct carcinoma. Interestingly, cytocidal effect of talaporfin after PDT was significantly higher than that of porfimer sodium with lower phototoxicity associated [225]. In another pilot study for treating locally advanced pancreaticobiliary malignancies using Photolon^®^, endoscopic ultrasonography-guided PDT in four patients was conducted with promising preliminary results which encouraged authors to do larger Phase I/II clinical trials to assess the local treatment in patients who are poor candidates for surgery or have unsuccessful chemoradiotherapy [226]; Phase II of the trial is ongoing. A recently started Phase IIa clinical trial, is evaluating deuteporfin PDT plus stenting versus stenting alone as treatment for unresectable advanced perihilar cholangiocarcinoma (Clinicaltrials.gov ID: NCT02955771).

Overall, the efficacy of PDT in patients with non-resectable biliary cancer has been demonstrated in a number of studies. PDT can be used without losing efficacy over multiple doses and in general is well tolerated by the patients. However, data regarding the use of PDT in neoadjuvant setting (i.e., prior to surgery) is limited and further studies should be conducted to assess the efficacy of PDT in this setting [227].

#### 3.2.6. Pancreas

Similar to bile duct cancer and lung cancer, pancreatic cancer is often diagnosed as late stage disease, making treatment with curative intent difficult. Tumor heterogeneity on both cellular and genetic level leads to resistance against RT and CT. Besides the variety of mutated oncogenes often found in pancreatic tumor cells, there also is a population of cells present with stem cell like properties that potentially makes them therapy resistant [228]. Consequently, pancreatic cancer is a very deadly disease and even with the increased understanding of its biology, it remains difficult to treat. Due to late diagnosis, less than 3% of the patients are eligible for surgery with curative intent [229]. CT is an option for inoperable pancreatic cancer but is associated with high morbidity and although significant, only minor improvement of survival [230]. Owing to its mode of action, PDT circumvents most tumor therapy resistance mechanisms and therefore is a modality with potential in effectively treating pancreatic cancer.

The first clinical trial of PDT in the treatment of locally advanced PC was conducted in 2002 using mTHPC [231]. PDT could produce tumor necrosis in all patients with low morbidity and mortality. The results of Phase I trial suggested applying PDT for localized cancers in patients who are not surgical candidates. Verteporfin^®^ was evaluated in a Phase I/II dose escalation study where it was shown that tumor reduction could be achieved safely with low morbidity. This study is still ongoing (EudraCT ID: 2006-004097-28), but PDT seemed feasible and safe for the treatment of pancreatic cancer and further trials are warranted [232].

#### 3.2.7. Bladder

With nearly 430,000 new cases diagnosed worldwide in 2012, bladder cancer is the ninth most common cancer [130]. Even though mortality is low, recurrence and disease progression rates are relatively high [233]. PDT is seen as an attractive alternative to CT often applied in bladder cancer.

In a pilot study, the effectiveness and safety of PDT using Radachlorin^®^ in patients with high grade, nonmuscle invasive bladder cancer, which was refractory or intolerant to bacillus Calmette-Guerin therapy, was shown after complete transurethral resection with no severe adverse effects [234]. In a Phase I study with patients suffering from intermediate or high-risk urothelial cell carcinoma, HAL-PDT was safe and appeared effective, but efficacy needs to be further assessed in larger cohorts [235]. A currently ongoing study is investigating the use of hypericin in patients with muscle invasive UCC (EudraCT ID: 2007-001302-25). While recent studies in the bladder are promising, further comparative trials in larger patient samples are needed to prove the efficacy of PDT.

#### 3.2.8. Female Reproductive Tract

The most common reproductive cancers in women are uterine, cervical, ovarian, vaginal and vulvar cancer. Reproductive cancers are often treated with surgery, chemotherapy, hormone therapy or radiation. Most standard therapies are known to have side effects and increase the risk of pregnancy related AE [236]. PDT has therefore been assessed as an alternative for its superficial therapeutic action and minimal side effects.

PDT using Photofrin^®^ was investigated in a Phase II trial for peritoneal carcinomatosis and sarcomatosis, including ovarian cancer [237]. Serious side effects were reported, such as intra-abdominal bleeding, bowel fistulae or anastomotic leak and ileus. In a group of 100 patients, they were unable to show complete responses or long-term tumor control. The heterogeneity in photosensitizer uptake, tissue optical properties, and tumor oxygenation, contributed to the lack of efficacy.

Persistent infection with the sexually transmitted, oncogenic human papillomavirus (HPV) is responsible for nearly all incidences of cervical cancer and high grade squamous intraepithelial lesion of the vulva (vulvar HSIL, formally denoted vulval intraepithelial neoplasia, VIN). Following infection, the cervical or vulval epithelium might develop into cervical intraepithelial neoplasms (CIN) or vulvar HSIL. Spontaneous regression often occurs in grade 1 CIN (CIN1) while for non-regressing CIN2 and CIN3 and vulvar HSIL treatment is recommended.

Wang and co-workers investigated PDT with the topical use of 5-ALA [238]. Treatment could be accomplished in a repetitive manner with 1 week interval, on an outpatient basis, and no severe side effect was described. All five patients in this study (four with CIN2 and one with CIN3) did not show recurrence of the lesions up to 9 months after PDT.

A Phase II clinical trial is currently assessing the effectiveness of PDT with ALA for the treatment of patients with HPV + low grade cervical intraepithelial neoplasiais (Clinicaltrials.gov ID: NCT02631863).

Positive results were described with PDT using Photolon^®^, a combination of chlorin e6 potassium salt and low-weight polyvinylpyrrolidone, in a study with a total of 112 patients with CIN2 and CIN3 [239]. A complete regression of the neoplastic lesions was documented, proven by morphological examinations, in 104 of the 112 (92.8%) treated women. Recurrance rates of vulvar HSIL lesions are high and similar for different treatment modalities (40%–48%) as shown by Hillemans et al. for treatment with PDT, surgery or CO_2_ laser [240]. Using the heamatoporphyrin derivative Photogem^®^ complete response rates of 71.4% (10/14) for vulvar HSIL lesions was accomplished at 1 year follow-up [241]. Normal anatomy and sexual function were preserved. However, three patients suffered from adverse effects of which two experienced skin photosensitivity problems and one perineal pain.

Several studies have employed 5-ALA derivatives, also topically, which are of interest due to the few side effects and minimal tissue penetration. Hexaminolevulinate (HAL) and methylaminolevulinate (MAL) are examples of such derivatives, more lipophilic than 5-ALA, thus with higher bioavailability. Hillemanns and coworkers investigated the pharmacokinetics and tissue selectivity of HAL in CIN lesions and concluded that this was a promising molecule for fluorescence diagnosis [242]. For PDT, the authors recommended a 10 mM dose of HAL and a 300–540 min interval between topical administration of HAL and light application in CIN. Such parameters were employed thereafter to investigate the feasibility and response rate in patients with CIN 1–3 lesions [243]. Patients with CIN 1, 2 and 3, (seven, 10 and seven patients, respectively) had remission rates of 71%, 50% and 71%, respectively, thus with a complete response for 15 out of 24 patients (63%). The authors suggested that HAL PDT can be performed on cervical lesions in a non-invasive and repeatable manner, with minimal side effects and, importantly, preserving cervical function. Later, a dose finding study showed that 40 mM HAL, when combined with a short DLI (3 h), gives promising results with high response rates (83%) and low morbidity, especially for lower grade CIN [244]. The efficacy of HAL for CIN was confirmed in a controlled study using a similar protocol for CIN1 patients. Due to the high rate of spontaneous HPV clearance, no significant difference was seen in the eradication of high risk HPV between the PDT and the control groups. Nevertheless, HAL-PDT showed high clearance rates and a favourable safety profile compared to surgery [245]. A follow-up study showed no significant effect of HAL-PDT compared to placebo groups for CIN1/2, but a significantly improved response was seen compared to placebo in CIN2 patients, which sustained towards the 6-month follow up. HAL demonstrates a beneficial curative effect compared to placebo for CIN2 patients and Phase III confirmation was recommended [246].

Importantly, a study confirmed that PDT with HAL and MAL does not induce any damage to normal cervical tissue [247]. Namely, no macroscopic changes of the cervix were observed and histological analyses half a year after PDT revealed no signs of apoptosis, necrosis, irritation, vascular changes, nor fibroses. This is certainly very relevant as cervical insufficiency or stenosis could have implications on pregnancy or cervical cancer screening.

Altogether, PDT shows to be safe and effective to treat cervical cancer when employing ALA or its derivatives. Further trials will confirm its potential as an alternative to standard procedures.

#### 3.2.9. Prostate

Prostate cancer is the second most commonly diagnosed cancer in men with 1.1 million diagnoses worldwide in 2012. It caused over 300,000 deaths that same year [130]. Incidence is rising with the availability of prostate specific antigen (PSA) testing and subsequent biopsy to help diagnose early. PSA now acts as a surrogate endpoint and as a biomarker of therapy response and disease recurrence. Most commonly used therapies are RT, prostatectomy and hormone therapy. Recurrence is a problem with RT and surgery, while hormone therapy is not curative. Moreover, incontinence and impotency are common side effects [248]. The alternative therapeutic approach to those just mentioned could be PDT which has been the subject of a number of studies.

In a group of men undergoing radical retropubic prostatectomy, Sulman and coworkers evaluated the selectivity of 5-ALA to prostate malignant cells. In their study, Protoporphyrin IX (PpIX) fluorescence was selectively concentrated in prostate cancer cells [249] and no fluorescence was observed in the stroma or in benign tissues of prostate in none of the patients.

In a Phase I clinical trial, after 10 sessions of PDT using mTHPC in early diagnosed patients with prostate cancer, PSA decreased by 67% and MRI scans showed tumor necrosis [250]. In another Phase I clinical trial, the safety and feasibility of using WST11 (Tookad^®^) as an intravascular photosensitizer was evaluated in patients with recurrent localized cancer after external beam radiation therapy which showed promising results [251]. Furthermore, in a Phase II study conducted by the same group, the efficacy of Tookad^®^ was assessed in patients and resulted in complete biopsy-negative response after applying high light doses [252]. More recently, in another Phase II clinical trial, Tookad^®^ was well tolerated and effective after i.v. administration and patients were free of cancer assessed by negative prostate biopsy [253]. The promising results led to a Phase III trial.

Currently, two Tookad^®^ trials are ongoing, one is investigating the safety and QoL for localized prostate cancer. This study will also assess safety and efficacy for recurrent and persistent prostate cancer (EudraCT ID: 2008-000876-26). The second trial has similar endpoints but will also focus on the difference between low and high volume prostates (EudraCT ID: 2009-012809-19). Tookad^®^ has shown promising results in earlier small scale clinical trials as reviewed by Kawczyk-Krupka et al. (2015) [254]. In summary, PDT has proved to be an effective therapeutic approach for prostate cancer, possible to apply at different stages of the disease, and can be combined with surgery.

#### 3.2.10. Brain

Surgical resection is the most common treatment for brain tumors and is routinely combined with post-operative CRT. In many cases however, the possibility of brain tumor resection is restricted due to its sensitive position and to preserve healthy brain tissue. As such, the tissue sparing characteristic of PDT could offer an alternative to conventional methods for aggressive and difficult to treat tumors such as Glioblastoma multiforme. Alternatively or in combination, the fluorescence of the PS has been used to guide the resection of tumors, known as fluorescence guided resection (FGR). Here, 5-aminolevulinic acid (ALA, typical dose 20 mg/kg), which converts into the active fluorescent molecule protoporphyrin IX (PpIX), has been mostly used. PpIX is excited by light of 357–440 nm wavelength, which although not appropriate for deep tissue imaging, is sufficient for superficial detection. Stummer and colleagues reported a more complete resection of tumors that led to improved progression–free survival in patients with malignant glioma [255]. A similar observation was documented in a study where ALA FGR was followed by interstitial PDT [256]. Here the cumulative 6 months progression free survival rates were 41% and 21%, for FGR combined with PDT, compared to PDT alone.

In a different study, the combination of 5-ALA and Photofrin^®^ was employed for FGR, followed by repetitive PDT [257]. This controlled trial showed a survival advantage (52.8 weeks of mean survival in the study group of 13 patients, compared to 24.6 weeks in the control group of 14 patients) without added risks to the patients with glioblastoma multiforme. Muller and colleagues reported on the results of Photofrin^®^ PDT alone and although the encouraging results, the authors suggest that higher light doses than the ones employed in this study may be required for better responses [258]. Another study employed Foscan^®^ as alternative PS for FGR followed by intraoperative PDT [259]. In this context, prediction of tumor was made with 90.7% accuracy and the detection of the PS proved to be of added value as 75% of tumor resections were radical, compared to 52% in the control group. The mean survival for patients that had FGR followed by PDT was 9 months, compared to 3.5 months of the control group, which was a significant improvement.

A preliminary clinical study tested the safety and efficacy of talaporfin-PDT for patients with completely removed, subtotally removed or partially removed gliomas, which were either recurrent or newly diagnosed. PDT had positive results in newly diagnosed tumors with high rates of tumor response and prolonged patient survival. With recurrent gliomas, PDT performed considerably worse with low tumor response, possibly due to inadequate penetration of tissue at the light-dose regimen applied in this study [260]. A follow-up study further examined the efficacy and safety of intraoperative talaporfin for recurrent or newly diagnosed glioblastoma multiforme (GBM) [261]. PDT considerably increased both overall and local median progression free survival and overall survival was prolonged compared to these parameters after CRT or FGR as reported in literature [255,262]. Again, results were better for newly diagnosed gliomas [261].

Currently two clinical trials are ongoing: one is a Phase I study assessing PDT for recurrent malignant brain tumors with poor prognosis using Photofrin^®^. Here, overall survival for 3 years post PDT treatment will be followed (Clinicaltrials.gov ID: NCT01682746). The other is a phase II clinical trial investigating PDT with Photofrin^®^ for recurrent high grade gliomas in adult patients (Clinicaltrials.gov ID: NCT01966809).

These clinical studies show PDT can be a beneficial addition to glioma treatment modalities, especially for newly diagnosed tumors.

#### 3.2.11. Other Organs

There are a number of other organs in which PDT has been investigated for clinical use. Talaporfin-PDT has shown to be an effective palliative treatment for a patient suffering from inoperable gastric cancer [263]. For the precancerous condition anal intraepithelial neoplasia, PDT using different photosensitizers has been investigated. mTHPC has shown to be only partially effective, whereas the results for ALA and Photofrin^®^ are encouraging, so much that the investigators plan a second pilot followed by a multicentral trial [264,265]. PDT for treating cancers of the eye is based on the treatment protocols for macular degeneration using verteporfin-PDT. In most cases, tumor thickness is decreased and visual recovery is demonstrated [266,267,268,269].

### 3.3. Current Limitations of PDT in the Clinic

As discussed in the previous section, PDT proves to be effective in inducing tumor responses as well as improving patient survival and QoL. Efficacy is seen when PDT is part of a multimodal approach, is used as a first-line treatment for premalignant or early disease and as standalone palliative treatment. Even though PDT shows great potential, there are still some limitations that prevent a firm position for PDT in standard care regimen of cancer. When reviewing the clinical trials and studies done over the last ten years, some general problems become evident.

A major problem is related to the adverse events (AEs) associated with PDT. With systemically administered PSs, especially of the first generation, skin photosensitivity is one of the most common AEs (see Supplementary Table S1). Patients have to avoid sunlight and strong artificial light for weeks, which is highly undesirable when they are nearing the end of life. Another AE often reported is pain. The main mechanism in PDT induced pain has yet to be elucidated, but several studies have found some predictors of pain. The biggest predictors appear to be the size of the treated area while location, PS type, lesion type, gender, age and light protocol have also been mentioned [270]. Several strategies of pain management have been tested but none fully relieved PDT induced pain [270]. The occurrence of AEs like inflammation, fever and nausea are typically location dependent but are often successfully managed with medication.

Another drawback is the decreasing efficacy of PDT for larger lesions, especially with first generation PSs. Due to inadequate tissue penetration of light or PS, bulky or deep seated tumors are difficult to treat with PDT. Especially evident with Photofrin^®^ and 5-ALA, the limited penetration of the appropriate light prevents sufficient depth of tumoricidal action [168]. Even second generation PSs perform less in larger lesions in which case surgery is more effective [167,207]. The most effective PSs tend to be hard to dissolve due to hydrophobicity and can form aggregates that have trouble penetrating tumor tissue [44]. Tissue penetration remains a topic of concern for PDT efficacy, especially when compared with systemic chemotherapy, radiotherapy or surgery. Insufficient tissue penetration could also be due to inappropriate PS and light dosimetry as a consequence of suboptimal protocols since there are very few generally accepted protocols available.

Besides larger lesions, PDT is also not indicated for metastasizing tumors. Almost all clinical studies exclude patients with tumor metastasis as it is almost impossible to reach those tumors with light. Metastasis remains one of the largest challenges in cancer therapy and PDT is no exception.

Tumor recurrence is often reported in clinical trials, probably due to inadequate tumor eradication. Not only insufficient penetration, but also the presence of PDT resistant tumor tissues due to hypoxia probably adds to the chance of recurrence. Although the reason for recurrence often lies beyond the scope of clinical trials, the importance of pre-existing hypoxia in cancer therapy outcome is well known [103,108].

## 4. PDT in Preclinical Studies

Here, preclinical studies of the last five to ten years are discussed that are investigating approaches to overcome the obstacles seen in recent clinical trials.

### 4.1. Targeted PDT

One of the more interesting fields in preclinical PDT studies is targeting. By improving PDT localization it can be expected that there will be increased efficacy in tumor destruction and fewer side effects related to off target localization [271]. Additionally, more effective targeting would also decrease the PS dosage needed, further limiting side effects. Targeting can be achieved on tissue, cellular and sub cellular level in PDT. Selectively addressing tumor tissue is particularly important in reducing side effects. As of now, (partial) tumor selectivity is mostly dependent on the passive EPR effect and the localized PS activation by specific tissue illumination. Actively targeting tumor cells can be achieved by a variety of approaches such as the conjugation of receptor ligand peptides, monoclonal antibodies, carrier proteins, carbohydrates, or loading into targeted nanoparticles (NP) [272,273,274,275].

Nanoparticles have been employed as packaging system to deliver PS preferentially to tumors, making use of the passive targeting by EPR effect, with the intention to decrease the general association to serum proteins and their general distribution, further protecting normal tissues [276]. In these approaches, the large loading capacity of such nanoparticles renders them very attractive for efficient delivery of PS to the target tissue. Moreover, for very hydrophobic PS these also act as a solubilizing vehicle. One of these approaches will soon be evaluated in a clinical trial, employing liposomal benzoporphyrin derivative, or verteporfin, for PDT on patients with primary breast cancer (ClinicalTrials.gov: NCT02872064).

Nanoparticles have been ameliorated in preclinical studies by the incorporation of targeting ligands/moieties on their surface. These moieties ensure the active targeting, by mediating interactions with particular proteins present on the surface of cancers cells. One example of a well explored interaction for receptor-mediated drug targeting is the Folate receptor (FR). This receptor is lowly expressed or absent on normal or healthy tissues but consistently expressed in several types of cancer and therefore an interesting therapeutic target [277,278]. The conjugation of the FR ligand, folic acid (FA), to micelles containing Foscan^®^ showed significantly increased tumor uptake, increased intratumoral Foscan^®^ concentrations and consequently enhanced tumor inhibiting effects in a xenograft mouse model. These effects were FR dependent as no differences in anti-tumor effect were seen between free and encapsulated Foscan^®^ in FR negative tumors. In this case, encapsulation of Foscan^®^ in untargeted micelles did not increase efficacy. The conjugated micelles significantly decreased skin phototoxicity and decreased the drug dosage needed to achieve similar efficacy. Both effects would decrease the side effects compared to free PS as lower dosage would also result in faster clearance [279]. Another interesting receptor is the epidermal growth factor receptor (EGFR) which has been the subject of numerous targeted cancer therapies. As with FR, EGFR expression is also upregulated in various types of cancer [280]. EGFR targeted micelles loaded with the second generation PS phthalocyanine-4 (Pc 4) showed increased tumor cell selectivity and rapid intracellular PS uptake. This resulted in an enhanced tumor cell specific photokilling effect with short DLIs. Moreover, this particular nanoconstruct allows multifunctional modifications such as the addition of imaging agents or drugs [281]. EGFR targeting proved effective in vivo as a human tongue carcinoma xenograft mouse model showed increased tumoral uptake, enhanced PDT efficacy and prolonged anti-tumor effect of the EGFR targeted PS loaded micelles compared to untargeted micelles [282].

Alternatively, the direct conjugation of PS to such targeting moieties has been explored. For instance, FA has been conjugated to a Foscan^®^-like PS via poly (ethylene glycol) (PEG), which increased its tumor selectivity by a factor of two compared to non-conjugated Foscan^®^. The conjugate achieved a tumor-to-healthy tissue ratio of 5:1 in nude mice xenografted with FR-positive tumors [283]. In a different study, the chlorin based PS pheophorbide-a was conjugated to several ligands to increase its tumor cell specificity [284]. The used moieties were FA, the CRGDLASLC peptide which binds to the αvβ6 integrin which is overexpressed in head and neck squamous cancer cells, the cRGDfK peptide that binds to αvβ3 integrins which are important in tumor angiogenesis and metastasis, and leuprorelin which is a ligand for the luteinizing hormone-releasing hormone receptor, which is overexpressed by ovarian, breast and prostate cancer cells. The pheophorbide-a conjugates were favourably taken up by cells that were overexpressing the corresponding receptors compared to the receptor negative cells. The FA- and especially CRGDLASLC-conjugates showed a receptor specific photokilling effect. Interestingly, the cRGDfK- and leuprorelin conjugates did not show a difference in photodynamic effect between the receptor positive and negative cells, despite the increased uptake, indicating that increased selectivity alone does not guarantee enhanced efficacy [284].

Instead of receptor ligands, conjugation of monoclonal antibodies (mAb) to PSs has also been employed to enhance tumor specificity. This approach, referred to as photoimmunotherapy, was first mentioned in 1983 by Mew et al. who conjugated HPD with a mAb to a tumor specific antigen on myosarcoma cells [285]. This increased HPD specificity and directed tumor cell killing both in vitro and in vivo. Results showed that the mAb conjugated HPD specifically targeted the antigen presenting tumor cells in vivo and significantly increased tumor inhibition after illumination. By increasing the DLI, the specific targeting effect was even more pronounced as the mAb accumulated in the tumor over time [285]. Since then several mAbs have been approved for clinical use in cancer therapies which could also be used for PIT [286]. Again EGFR proves successful as several studies have investigated the use of anti-EGFR mAbs for PS targeting [287,288,289]. In a proof of concept study, verteporfin was coupled to an anti-EGFR mAb. Antibody conjugation increased verteporfin uptake by EGFR expressing A431 cells compared to untargeted verteporfin. In vivo, increased concentrations of anti-EGFR-verteporfin were detected in EGFR positive tumors compared to EGFR negative tumors or healthy tissue. Moreover, tumor inhibition and mice survival after irradiation was increased with targeted verteporfin compared to untargeted verteporfin or controls indicating mAb targeting could both increase selectivity and therapeutic effect [287].

The commercially available mAbs trastuzumab and panitumumab, which are directed against HER2 and EGFR respectively, were used to provide selectivity to the water soluble PS IRDye700DX (IR700) [288]. This phthalocyanine dye has an ester that allows covalent conjugation of mAbs. It also absorbs light at a longer wavelength (689 nm) than most PSs such as Photofrin^®^ or Foscan^®^, increasing depth of light penetration. In vitro studies showed that the mAb-IR700 conjugates only induced death in cells expressing their respective receptors. Moreover, the conjugates needed to be bound to the cell to have photocytotoxic effect as free IR700 or unbound conjugates did not induce cell death. In vivo studies using xenografted mice bearing both receptor-positive and -negative tumors showed receptor dependent IR700 accumulation and tumor eradication [288]. Currently, a Phase I trial with 2 parts is ongoing, where the mAb cetuximab targeting EGFR is conjugated to the PS IR700 for PDT of patients with recurrent head and neck cancers (ClinicalTrials.gov: NCT02422979). The first part is a drug-escalation study, while the second is a light dose escalation study. The outcome of this trial will certainly influence further evaluation of these or other targeted PDT approaches in the clinic.

A known restraint of antibody-targeted therapy is the insufficient intra-tumoral distribution of the therapeutics [290]. To address this problem a mixture of differentially targeted PS conjugations was tested. IR700 was conjugated to either anti-EGFR (panitumumab) or anti-CD25 (basiliximab). In vivo studies showed a better intra-tumoral distribution of PS and a beneficial anti-tumor effect with improved survival when combining Pan-IR700 and Bas-IR700 conjugates compared to either conjugate alone. The presence of a “binding site barrier” leading to heterogeneous intra-tumoral distribution can be overcome with combinations of antibody guided PSs, further increasing PIT efficacy [289].

In spite of its therapeutic potential, conjugating PSs with relatively large mAbs will increase the PS half-life which is an undesired characteristic leading to prolonged photosensitivity [273]. As such, it was attempted to use smaller antibody fragments [291,292,293,294,295]. Recently, nanobodies (NBs) have been evaluated as an alternative for mAbs. NBs only consist of the variable binding domain of heavy chain antibodies that, despite their size, can specifically bind antigens such as EGFR. Oliveira et al. [296] directly compared anti-EGFR NBs with mAbs (cetuximab) as targeting probes for a near-infrared fluorophore used to visualize tumors. In vivo, the NB conjugates showed faster tumor accumulation and better intra-tumoral distribution compared to cetuximab [296]. The same nanobody was later evaluated for targeted PDT, directly conjugated to IR700 (PS) [297]. Incubating cells with no, or different expression of EGFR with the NB-PS conjugates showed a correlation between EGFR expression and NB binding based on PS fluorescence. No binding to cells was observed of free PS or R2-PS (R2 being a negative control nanobody). Subsequently, cell death was only observed in cells with high EGFR expression, mediated by NB targeting EGFR conjugated to the PS. An in vivo proof of principle study was conducted with these NBs conjugated to PS, where light was applied 1 h post administration of the conjugates, in a model of oral squamous cell carcinoma [298]. This approach led to extensive tumor damage (approx. 90% tumor necrosis) with minimal toxicity to the surrounding tissues, thereby highlighting the selectivity of this approach. Further evaluation of this NB targeted PDT will reveal the full potential of this, thus far promising approach.

Altogether, conjugating PSs to mAbs or NBs improves PDT selectivity and can be crucial in mediating cell killing efficacy of hydrophilic PSs. It also enhances PDT efficacy so PS dosage can be lowered, thereby reducing unwanted systemic side effects.

Intracellular targeting of PS can be achieved by simultaneously targeting tumor cells and mitochondria. This multimodal approach first actively targets tumor cells and after internalization actively localizes the PS towards mitochondria to promote PDT induced apoptosis. While PS usually localize towards mitochondrial membranes due to electrostatic interactions, it is also possible to target mitochondrial proteins [299]. The mitochondrial translocator protein (TSPO) has been used as a target for PDT [300]. TSPO is expressed in healthy tissue but overexpressed in multiple cancers possibly facilitating tumor cell selectivity. Indeed, by conjugating the TSPO ligand I-PK 11195 to HPPH, its tumor selectivity was enhanced and skin photosensitivity was reduced in xenograft colon- and breast cancer mouse models [300]. A similar attempt was made with IR700, which was attached via a linker to the TSPO ligand DAA1106 to create the targeted PS IR700-6T. In vitro results showed IR700-6T to selectively induce apoptotic cell death in TSPO-positive but not in TSPO-negative breast cancer cells. It also significantly inhibited tumor growth compared to IR700 in vivo in a xenograft breast cancer mouse model [301].

### 4.2. Light Dosimetry and Treatment Planning

The advantage of only being activated with the appropriate delivery of light is also one of the shortcomings of PDT as delivery of light is challenging in deep-seated, bulky, solid tumors [302]. As described earlier, the photodynamic effect of first generation Photofrin^®^ isn’t enough to eradicate larger lesions [140] or to reach the tumor tissue in the outer wall of the bile duct [219]. In fact, PDT was initially only indicated for smaller sized lesions but second generation PSs show improved efficacy with larger lesions increasing the range of PDT utility [142,202,207].

There are several ways of improving light tissue penetration. One way is to develop PSs with absorption of light at higher wavelengths, preferably NIR. The beneficial effect of this property has been well known with the increased tissue penetration and efficacy of second generation PSs such as Foscan^®^, verteporfin and talaporfin, compared to Photofrin^®^. Nevertheless, even though these PSs are able to penetrate the tissue deeper, some of the larger tumors still can’t be fully covered by sufficient light to achieve the desired PDT effect. Therefore, it is often attempted to improve light delivery with interstitial PDT (iPDT) to achieve larger areas of necrosis. iPDT is advantageous over superficial PDT as the insertion of (several) optical fibres can deliver a more uniform and deeper distribution of light throughout the tumor. Adequate light dosimetry is crucial as insufficient light delivery might leave residual malignant cells leading to tumor recurrence while too much light might penetrate too deep and affect healthy tissues. The technology for this modality has improved over time with continuous development of new light sources, optical fibres and the expanding knowledge of light dosimetry [303]. iPDT has been used in both preclinical and clinical settings and shown satisfactory results [133,232,304,305,306,307,308].

A preclinical study using intramuscular established tongue tumors in a xenograft mouse model, compared HPPH-iPDT with superficial HPPH-PDT for deep-seated bulky tumors otherwise not indicative for PDT. Compared to superficial PDT, iPDT had increased photodynamic effect as seen by MRI imaging and histological analysis of the tumors. iPDT induced significantly more tumor cell necrosis and vascular damage [309]. The potential of PDT for deep-seated tumors has also been proven in a clinical setting where patients with tongue based SCC, unsuited for conventional therapy, were treated with iPDT. Ultrasound guided placement of fibres was used in combination with Foscan^®^ administration. The majority of patients reported improved breathing, swallowing and speech due to tumor reduction, illustrating the potential of iPDT for lesions otherwise untreatable by superficial PDT [308].

Better placement of the optic fibers using improved visual guiding techniques offers the ability to optimize (interstitial) PDT efficacy for otherwise hard to reach tumors. The combination of PDT with such navigational techniques is finding its way into clinical trials. For example, the use of Electromagnetic Navigation Bronchoscopy to improve the usability of Photofrin^®^ in lung cancer (Clinicaltrials.gov ID: NCT02916745).

In the last five years, significant effort was put into improving iPDT for several cancers. It is attempted to create a way of calculating the optimal light dose needed for the desired iPDT effect and ultimately to find a way of real-time assessing the light dosage and being able to appropriately configure the light source during the intervention. The first step of dosimetry is based on mathematical models that account for tissue optical heterogeneity. A new algorithm was found called the Kernel calculation, which improves computational efficiency and could be used in real-time light fluence dosimetry [310]. These computational algorithms can be used in interstitial diffuse optical tomography (iDOT) systems that can characterize optical parameters of biological tissues [311]. Using silicon based prostate models called phantoms, the iDOT is used to measure and calculate desired iPDT light dosage based on the reconstructed spatial optical properties [312]. The accuracy of phantom or turbid media based measurements and algorithms have been compared to in vivo measurements and appeared robust enough for clinical use [313]. The iDOT or other similar systems would be used in a clinical setting by measuring the penetration of its light sources in the lesion and calculate optimal light dosimetry by measuring tissue light diffusion. Using this data, the optimal iPDT protocol could be reconstructed, determining the dose plan, the number and position of light sources, and adjusting these parameters in real time. Such a system was already tested in a clinical setting and results showed its use is feasible but needs improvement [314]. This would ultimately lead to a robotic platform that could both measure and calculate the optimal light dosage but also deliver an adequate light regime by optimizing the positioning of multiple sources and performing dosimetry in real time during treatment [315]. Intra-operative dosimetry is increasingly used to ensure optimal light dosimetry in clinical trials (Clinicaltrials.gov ID: NCT02662504 and NCT02464761). Improved means of measuring and calculating light and drug dosimetry, and light source placement, combined with the use of automated light dosimetry and real time controlled iPDT, can improve PDT efficacy for hard to reach or bulky tumors.

### 4.3. Battling Hypoxia

Although not often mentioned as a limitation of PDT in clinical trials, tumor hypoxia is an obstacle that still needs to be overcome. As mentioned earlier, hypoxic regions in solid tumors are protected from oxygen dependent PDT. These low oxygen areas are already present pre-PDT [316] or emerge due to PDT oxygen consumption or vascular collapse [54]. Hypoxic tumor models show highly variable and non-reproducible results following PDT [317]. Still lots of effort is invested into PDT-related oxygen/hypoxia studies to increase the knowledge on oxygen depleting mechanisms, improve in vitro models and optimize in vivo PDT outcome prediction [318,319,320,321].

There are strategies to overcome PDT induced hypoxia such as lower light fluence rates or fractionated illumination to prevent oxygen depletion and allow sufficient tissue re-oxygenation, but results vary and such approaches would not resolve pre-existing hypoxia issues [322,323,324,325]. However, an interesting proof of concept study wanted to take advantage of fractionated PDT by creating a PS that could produce singlet oxygen during the dark periods [326]. A PS was constructed with a 2-pyridone ring that will produce singlet oxygen upon irradiation, but can also “store” some of the singlet oxygen by converting to 2-pyridone-endoperoxide. During the dark phase, 2-pyridone-endoperoxide will undergo thermal cycloreversion releasing singlet oxygen in the process. Indeed, the conjugate was able to have continuous release of singlet oxygen during both the dark and light phases of fractionated PDT possibly circumventing PDT induced hypoxia [326].

It is also possible to take advantage of PDT induced hypoxia by co-treatment with a therapeutic that is activated under low oxygen conditions [327]. The pro-drug tirapazamine (TPZ) can be activated by various intracellular enzymes after which it can generate cytotoxic species. In normoxic conditions, the radical form of TPZ is rapidly converted back to the ineffective parent molecule. TPZ is currently used in a clinical trial as part of a multimodal cancer therapy approach (Clinicaltrials.gov ID: NCT02174549). By combining PDT with TPZ in a micellar structure the researchers attempted to make use of PDT induced hypoxia to further damage tumor cells by TPZ radicals. As seen in vitro, PS carriers with TPZ did more damage after irradiation than PDT alone, TPZ/PS without irradiation or PDT with free TPZ. Similar results were seen in vivo, where TPZ-PS synergistically induced the most pronounced tumor inhibiting effect compared to all other groups. Combining PDT with hypoxia dependent pro-drugs appears to be a promising approach to take advantage of PDT induced hypoxia [327].

Pre-existing tumor hypoxia can be circumvented by a variety of strategies such as improving tissue oxygenation prior to PDT. It is thought that the use of hyperbaric oxygen improves PDT efficacy [111,328]. Nevertheless, it shows only minor benefit and responses vary between protocols and types of cancer, illustrating the need for more and better (pre-)clinical studies [329]. Other strategies make use of PSs that are able to circumvent tumor hypoxia by efficiently producing radical species via type I reactions. The benzophenothiazine PS ethylamino-9-diethylaminobenzo-[a]phenothiazinium chloride (EtNBS) is able to directly interact with biomolecules and water to form lipid and protein radicals as well as hydroxyl radicals and superoxide species [330]. The tumor killing efficacy of EtNBS was compared to chemotherapy with carboplatin and PDT with a benzoporphyrin derivative (BPD) using in vitro grown 3D ovarian cancer tumor models. BPD was shown to predominantly localize to the periphery of the tumor nodules and treatment with either BPD or carboplatin, only affected the periphery of the nodules and left a surviving core of malignant cells. EtNBS was able to localize into the nodule core and after irradation effectively killed the core cells. EtNBS induced cytotoxicity via type II oxygen consuming reactions but could also remain cytotoxic under hypoxic circumstances via type I reactions [330]. The same group tried to improve EtNBS efficacy by creating a small library of derivative compounds that was screened using the same in vitro tumor model [331]. One derivative, EtNBS-OH, showed similar uptake, localization, and overall cytotoxicity as the parent compound. However, this derivative had a more pronounced nodule structure disrupting effect due to a more decentralized localization compared to EtNBS. Disruption of tumor structure leads to increased penetration of both oxygen and therapeutics thereby not only being able to be effective under hypoxic conditions, but also capable of improving both oxygen and therapeutics diffusion [331].

### 4.4. Optical Monitoring

In addition to the optimization of light source placement and treatment planning a significant amount of work, over the last 10 years, has been performed in the area of optical monitoring using both imaging and spectroscopy. This approach to PDT dosimetry can be used to identify when sub-optimal PS pharmacokinetics and hypoxia are obstacles to effective PDT. While much of this work has been performed in pre-clinical models, optical monitoring is now finding its way into clinical studies.

The absorption and fluorescence characteristics of PSs enable the opportunity to interrogate them using optical spectroscopy. The concentration of PS is an important parameter in the efficacy of PDT. Here differences in the uptake of photosensitizer in tumor tissue, between lesions and/or patients and differences between the uptake in tumor and the surrounding normal tissue are parameters that are critical to effective PDT. Monitoring photosensitizer pharmacokinetics (both temporal and spatial) is one of the most fundamental areas of PDT investigation and examples of these types of studies are too numerous to review. In this type of studies, it is important to carefully consider the path length of light in tissue if these types of measurements are to be quantitative. It is also important to note that absorption and fluorescence measurements should be interpreted with care. Fluorescence emission from fluorophores is influenced by their environment. There exists a complex relationship between the concentration of a chromophore and its absorption cross-section and fluorescence emission intensity. In vivo fluorescence (and to a lesser degree absorption) can be altered by many factors that include changes in quantum yield induced by changes in the microenvironment [332] photobleaching [95], biological compartmentalization, and alteration in binding and aggregation [333,334].

In-vivo absorption, and to a greater degree fluorescence spectroscopy, are often used to monitor pharmacokinetics in pre-clinical models. These techniques are much less often used in the clinical environment. This is unfortunate because they can potentially have the greatest impact. Measuring photosensitizer pharmacokinetics is not the only area in which photosensitizer spectroscopy can be utilized in guiding or monitoring PDT. As we have described above, PDT is a complex photo-chemical/biological process and is influenced by a wide range of parameters. Optical spectroscopy has been used to investigate the processes that occur during PDT. The process of progressive destruction of the photosensitizer during PDT, mediated by the generation of reactive oxygen species was recognized as an important factor in PDT dosimetry over two decades ago. This process termed photosensitizer photobleaching has since been investigated in numerous pre-clinical studies for many photosensitizers. Since these early studies, investigations utilizing photobleaching has led investigators to a greater understanding of the photochemistry that is underlying PDT and have been incorporated into dosimetric models for PDT [332,335]. Over this time period the understanding of the complexity of the role of tissue vasculature and the demand (and supply) of oxygen during PDT has increased dramatically. As we have reviewed above the important role of fluence rate on the photobiology that occurs during PDT and its relationship to PDT response is becoming increasingly clear [96]. In many circumstances the choice of clinical fluence rate is far above that that has been shown to be optimal in pre-clinical models. Again, it is important to highlight two points. First, it is critical to understand the mechanisms underlying the processes surrounding fluorescence photobleaching and how they relate to tissue response. These can be different for different photosensitizers and different for different environments. Second, just as for pharmacokinetic measurements it is disappointing that very few clinical studies have incorporated these types of measurements.

Reflectance spectroscopy can be used to interrogate the tissue before, during, and after PDT to monitor changes in the concentration of native absorbers. The predominant absorbers in the visible region of the spectrum in tissue are oxy- and deoxhemoglobin. These can and have been used to determine variations in physiological parameters such as blood saturation and blood content (volume). These types of techniques have been used in PDT by a number of investigators to monitor the vascular response to PDT [336,337]. Depending on the photosensitizer and its localization, the acute vascular response can be useful in predicting the overall response to PDT. These approaches are particularly important for predominately vascular-based photosensitizer such as BPD and Tookad. In this context it is important to consider blood flow in tissue undergoing PDT. Here, other novel approaches such as laser speckle imaging [338] and diffuse correlation spectroscopy [339] have been utilized to monitor blood flow. It is also possible to consider the use of other spectroscopy techniques such as Raman spectroscopy [340] and spectroscopic optical coherence tomography [341] but considering the complexity of these techniques they are not yet ready for implementation for guiding or monitoring PDT. More recently we (summarized in [342]) and others [343] have been working on approaches to recover the intrinsic fluorescence (i.e., that signal that is corrected for the influence of the tissue optical properties and can be used to recover the concentration of PS). Encouragingly, in a small number of centres, these types of measurements are being utilised in clinical PDT studies in inform PDT dosimetry and determine the relationship between optical monitoring and clinical outcome [344,345,346]. Spectroscopy is finding its way to the clinic and several currently ongoing trials use spectroscopy techniques to evaluate PDT dosimetry and efficacy (Clinicaltrials.gov ID: NCT02258243, NCT02367547, NCT02647151 and NCT02878382).

## 5. PDT in the Future

In the previous section, we have described a range of pre-clinical studies that have been performed to address some of the problems encountered in clinical trials, this section will focus more on PDT innovations that aim to improve PDT as a whole. With the considerable potential of PDT, a lot of effort has been expended into making it a standardized and widely accepted modality in the anti-cancer arsenal.

### 5.1. Theranostics and Multifunctional Nanocarriers

Combining the therapeutic and diagnostic properties of PSs, in a single, more patient specific approach, is an active field of research. These so-called theranostic agents are used to simultaneously diagnose and treat cancers and other diseases. PS are inherent theranostics to some degree as its fluorescent properties can be used to detect and define malignant tissues and possibly aid in evaluating treatment success, as mentioned earlier. The potential of PS based diagnosis was recognized with the approval of a 5-ALA derivative as a bladder cancer diagnostic [347]. However, the clinical use of PS based fluorescence imaging is often impaired by the limited penetration depth of activating light and aspecific localization leading to poor imaging contrasts between neoplastic and healthy tissues [4]. As with the therapeutic properties of PSs, diagnostic efficiency can be improved with increased selectivity for tumor cells by either adding targeting mechanisms or localized, cell specific activation of the PS [348].

Nanotechnology is often used to improve PDT selectivity and combine PSs with other diagnostics or therapeutics to form theranostics, whilst trying to retain the respective compound properties [349]. A wide range of NIR responsive nanomaterials such as gold nanoparticles, carbon nanotubes, graphene oxide and upconversion nanocrystals are used as a basis for theranostic applications in PDT [350]. These nanomaterials are able to absorb light in the region outside that of tissue autofluorescence, improving imaging quality. Moreover, due to their large surface area, they act as a scaffold on which PSs, targeting moieties, other diagnostic agents and other therapeutics can be assembled [350].

As said, PS fluorescence based imaging is limited to superficial, two-dimensional identification of tumors. In order to increase the utility of PDT based diagnostics, both older and recent publications show considerable interest in incorporating positron emission tomography/computed tomography agents [136,351,352,353,354] and MRI probes [355,356,357] in multimodal nanoparticles (extensively reviewed in [4]).

Recently the addition of other photo-activated therapeutics that improve the anti-tumor potential of PS based theranostics has often been investigated. The use of light activated photothermal therapeutic (PTT) agents can increase the overall cell killing effect compared to PDT alone, while the nanocarrier also holsters efficient imaging modalities for image-guided PTT/PDT [358,359]. These multifunctional nanoplatforms can become even more complex by incorporation of chemotherapeutics [355,360].

The interest in combining diagnostics and therapeutics in the field of PDT is illustrated by the vast body of literature of the last couple of years, describing the ideas and endless possibilities to create multi-layered PDT-based theranostics. These multifunctional modalities will improve the applicability of PDT and possibly strengthen its position in the clinic. Not only will nanomaterials provide a scaffold for both PS and targeting moieties, they will also enable the incorporation of imaging agents and other therapeutics to improve PDT efficacy and applicability [361]. Nevertheless, as with the mAb conjugated PSs, the increased size of such complex theranostic platforms might negatively affect PS circulation times and tissue penetration. Further studies are needed to show the applicability of such compounds.

### 5.2. PDT Induced Anti-Cancer Immunity

One of the hallmarks of PDT is the inflammatory response following treatment-induced tumor cell death. This response is also crucial for the development of anti-tumor immunity [362]. Few pre-clinical studies have shown the occurrence of tumor immunogenicity, control of distant disease and protection for further tumor challenges [76,363]. Not many clinical studies focus on this aspect of PDT but at least one case report mentioned the regression of untreated distant lesions after photodynamic therapy in the clinic [364]. A clinical study using PDT to treat vulval intraepithelial neoplasia patients showed that non-responders had down-regulated major histocompatibility complex 1 (MHC-I) expression and decreased T-cell migration compared to responders, hinting at the importance of the immune system in PDT outcome [365]. Considerable effort is put into understanding the mechanisms underlying tumor immune response and systemic anti-tumor immunity and exploring how to exploit them to improve PDT efficacy [69,366].

Earlier work mentions several ways of stimulating the immune system to improve anti-tumor immune-reactions after PDT. Administration of inflammatory cytokines such as TNF-α or macrophage colony stimulating factor or the local administration of pathogen-associated molecular patterns (PAMPs) such as bacterial or fungal components stimulated the immune-system and improved PDT efficacy [367,368]. The application of PAMPs to improve PDT induced tumor immunity is still being investigated. Peritumoral injection of CpG oligodeoxynucleotides, a TLR-9 agonist used in clinical trials to improve immunotherapy, resulted in tumor directed migration of primed DCs that show enhanced phagocytosis, maturation and antigen presentation to T-cells. When combined with PDT, this ultimately results in prolonged host survival in a metastatic murine breast cancer model compared to PDT alone [369]. Implementing CpG oligodeoxynucleotides in a nanocarrier together with a PS also had a synergistic anti-tumor cell effect in vitro using the same poorly immunogenic, metastatic breast cancer cell line [370]. Post-PDT administration of calreticulin, one of the key DAMP molecules, enhanced tumor response in immunocompetent but not immunedeficient mice [371]. Calreticulin was found to bind PDT damaged cells and stimulate migration and phagocytosis by DCs and macrophages. Combining PDT with the administration of PAMPs or DAMPs seems to improve PDT efficacy by stimulating immune cells that drive both the innate and adaptive immune system.

Interestingly, by combining a low-dose, immunostimulatory PDT regimen with a high-dose tumor-ablative PDT regimen, improved anti-tumor efficacy was seen [372]. The initial low-dose PDT regimens significantly increased neutrophil migration towards the tumor which wasn’t seen after high-dose PDT, probably as a consequence of vascular collapse. Following low-dose PDT with a high-dose regimen had a similar or improved effect on tumor ablation compared to high-dose PDT alone. In addition, the combination therapy led to improved local and metastatic tumor growth inhibition and enhanced anti-tumor immunity [372].

Rather than stimulating the immune system, it is also possible to inhibit immunosuppressive pathways to enhance PDT anti-tumor effect [373]. Promising results were acquired when PDT was combined with low-dose cyclophosphamide (CY), a chemotherapeutic used in the clinic which is able to deplete immunosuppressive regulatory T-cells (Tregs). Only after Treg depletion by CY administration, a long-lasting anti-tumor response could be established after PDT [373]. In a similar study, combining PDT with low dose CY led to long-term tumor regression in 90% of the treated mice, however all mice developed cancer after rechallenge [374]. Extending the duration of Treg depletion facilitated rejection of tumor rechallenge which was only observed after a second administration of CY prior to rechallenge. The presence of Tregs during PDT or tumor rechallenge suppresses the immune response and inhibits tumor immunity following treatment [374]. This could be translated to the clinic by using the already approved CY as anti-Treg therapy or other immunosuppressive therapies to improve initial PDT efficacy and to possibly prevent patient relapse after PDT.

Modulating gene expression by epigenetic reversal can also aid in enhancing PDT anti-tumor effect. Essential components in eliciting an immune response such as MHC I or tumor associated antigens (TAA) are often downregulated in cancers [375]. The importance of TAA in anti-tumor responses was elucidated when PDT treatment of P1A positive tumors elicited an epitope-specific immune response while treatment of P1A negative tumors did not [376]. By altering the DNA methylation using the clinically approved methyltransferase inhibitor, 5-aza-2′-deoxycitidine (5-aza-dC), the expression of MHC I and P1A can be restored, improving tumor antigen presentation and consequential immune cell recognition. This leads to increased P1A expression and presentation, an improved adaptive immune response post-PDT with long-term survival and anti-tumor immunity mediated rejection of rechallenge [377]. As the expression of other TAAs can also be modulated by epigenetic reversal, other epigenetic treatments and PDT combinations might prove effective for different tumors.

Additionally, PDT is used to generate therapeutic or prophylactic anti-tumor vaccines based on tumor cells or lysates obtained after ex vivo PDT [378]. PDT generated cell lysates proved more effective than lysates generated by UV or ionizing irradiation or freeze thaw cycles in inducing an immune response [82]. Following cell lysate vaccination, several studies reported stimulated DC migration and maturation, enhanced T-cell activation and tumor specific immune recognition leading to tumor growth inhibition, prolonged survival time and acquisition of resistance against rechallenge in mice [82,84,379]. Similar results were seen after administration of PDT treated whole tumor cells as a cancer vaccine [83].

An alternative to using PDT treated tumor cells or tumor cell lysates as vaccines is using live DCs. By exposing DCs to PDT treated tumor cells or lysates ex vivo, they can engulf the cells or free antigens and process them for presentation. Injecting DCs that were primed ex vivo with PDT induced cell lysates resulted in specific anti-tumor immunity against fully established solid mammary tumors in mice and prolonged survival time compared to injection of whole tumor cell lysates which had nearly no immunological effect [380]. However, this was probably due to the limited ER-stress induced by the used PS DH-I-180-3, which predominantly affects mitochondria. A recent study showed comparable immune responses after DC-based and lysate-based vaccination when the ER-specific PS hypericin was used. ER-stress induces the release and exposure of eat me signals such as calreticulin and HSP70, thereby promoting antigen recognition [379]. Efficacy of DC based vaccines has been confirmed using other PSs and other tumor models [381,382]. DCs that were primed with specifically isolated MHC I bound antigens after ex vivo tumor cell PDT were more effective in mediating an immune response [383]. By this method, the unspecific and possibly immunosuppressive antigens and cytokines are filtered from the cell lysate leaving a more efficient anti-tumor antigen pool to present to DCs. Administration of these primed DCs to tumor bearing rats created a more tumor specific vaccine compared to whole cell lysate primed DCs. Moreover, more efficient DC maturation, increased cytokine production and improved antigen-specific CTL responses in vivo were observed [383].

As the downregulation of immunosuppressive cells enhanced PDT-mediated anti-tumor immunity, the same approach was used in an attempt to improve anti-tumor vaccination induced immunity. Immunosuppressive Tregs and myeloid-derived suppressor cells (MDSCs) were depleted using CY and all-trans retinoic acid (ATRA) respectively before vaccination. Administration of either CY or ATRA, both before or after PDT-vaccination, significantly enhanced the vaccines’ therapeutic efficacy. An even greater effect is seen when the adjuvants are administered before and after vaccination indicating the role of immunosuppressive cells in attenuating immunostimulatory responses following PDT-vaccination [384].

## 6. Discussion

Apart from the clinical success of PDT in the skin using porphyrin pre-cursors and the promising stage III trials utilizing Tookad in the prostate, PDT is still an underutilized modality in the clinic, even though PDT has received regulatory approval as a cancer treatment for over 30 years. Considering recent clinical studies, PDT has proven to be successful as a curative intervention for premalignant and early disease and is considered an effective palliative intervention for late stage disease. Moreover, it can be used either pre-, intra- or post-operatively and in combination with other modalities to improve treatment outcome. Numerous studies report CR and improvement of QoL in patients with no other treatment options left. Due to its mode of action it can easily be used in conjunction with other treatments such as chemotherapy, radiotherapy and surgery.

The fact that PDT has not yet reached the status of “standard care” has several reasons. First of all, there is a lack of randomized controlled clinical trials of adequate power. When considering the clinical trials discussed here, most trials are small and implement PDT in a multimodal treatment to assess its beneficial effect, rather than directly comparing standalone PDT to standard care, preferably surgery. As such, studies have low power and PDT is less likely to be accepted as an alternative, instead of an addition to standard care. PDT is often used as a salvage treatment for recurrent disease or as palliative care for patients deemed incurable otherwise and even though it shows low morbidity and curative efficacy, in cases where standard care is contraindicated, it does not warrant the use of PDT as a first-line treatment.

In the field of head and neck cancer, direct comparisons between surgery and PDT are available and show similar treatment outcomes for smaller lesions [207]. Here, PDT is considered a good alternative for conventional therapy, partly due to the application of second generation Foscan^®^, which is a more effective PS than Photofrin^®^ that is often the only alternative for other indications. It should be noted that for thin lesions in the head and neck, oral laser microsurgical removal is often favoured over PDT in part due to the cost of Foscan^®^. Although PDT and surgery are similarly effective for early disease, cancers such as of the lung, pancreas and biliary tract are often diagnosed at a later stage with bigger or deeper lesions for which surgery still proves more effective.

Compared to CT or RT, PDT often proves to be an effective, safe alternative with lower morbidity as can be seen in the trials discussed earlier. When PDT is used as salvage treatment after failed RCT, often cases of CR are reported indicating PDT at times is even more effective [167,168,211]. Together with the significantly lower morbidity of PDT and the possibility of combining it with RT and CT, this suggests PDT should be considered as a viable alternative. However, surgery, CT and RT are established treatments that are available in most hospitals. PDT needs specific, relatively inexpensive equipment (compared to other infrastructure and equipment costs in for example a radiotherapy department) that is not widely available in the clinical setting. Normally the costs of light sources are either covered trial sponsors or are available in large PDT centres. This can be a barrier to widespread clinical use after clinical trials have ended. This does not only hamper the actual use of PDT for treatment, it also decreases the possibility for further clinical studies to establish better protocols. One of the most important factors to overcome is the difficulty in comparing clinical results performed in small studies between different centers, because of the use of different treatment protocols. As we have described, PDT dosimetry is complicated and the standardization of its implementation can be difficult. A concerted effort in this area is ongoing in a number of research groups. International clinical guidelines and a conscientious opinion are critical factors if PDT is to be more widely implemented. The problem of the establishment of better treatment protocols has also been highlighted by Moghissi, who has stated that the field of PDT research lacks commitment and funds. Commitment is needed from scientists creating new therapeutics, from the government to stimulate research and from clinicians to use and improve PDT in the clinic. This is also held back by the lack of funds [385,386]. Funding agencies rarely commit to funding continuing research clinical trials that may be necessary to optimize treatment protocols after a PS has been approved in an organ specific clinical setting.

Almost one third of the discussed recent trials and studies still used the first generation Photofrin^®^. Even though Photofrin^®^ proves to be effective in most studies, it is still held back by its side effects and its lack of light penetration, which does not encourage its use. Second generation PSs such as talaporfin, Foscan^®^ or HPPH have fewer side effects and increased efficacy. Studies using these PSs show PDT and surgery are similarly effective, even for larger lesions [142,207]. However, such studies are scarce and further research is needed to support these data. Yet, clinical trials that are currently ongoing in Europe seem to focus on dose-finding and assessing safety and efficacy data on newly discovered PSs rather than trying to establish a firm position for approved or well-studied second generation PSs. It would seem appropriate to try to obtain approval for talaporfin in Europe, which has shown great results in the clinic and multiple clinical studies in Japan. Confirming the efficacy of established PSs could aid in the translation towards the clinic. In addition, there is the need to establish standardized protocols for these PSs to further improve efficacy and reducing morbidity.

The clinical limitations of PDT are actively addressed in recent preclinical studies. By finding new PSs or improving tumor selectivity of known PSs it is attempted to further reduce the photosensitivity period, one of the major drawbacks of current PDT. Newer, second generation PSs already show improved photosensitivity profiles. Using targeting moieties such as antibodies or receptor ligands, it is attempted to direct PS accumulation and improve tumor tissue localization, thereby limiting off-target effects. Encapsulated PSs in targeted nanoformulations also show improved tumor localization and decreased skin photosensitivity. Targeting could also address the limited tissue penetration of current PSs. Better tumor infiltration and more homogeneous distribution of PDT compounds has been seen after the use of targeting strategies, resulting in improved tumor eradication [288,289].

Several approaches are investigated to improve PDT tissue penetration, in respect of light delivery. One widely applied method is iPDT which uses optical fibers to guide light deeper into the tissues thereby improving the depth of PDT action. iPDT is already successfully applied in the clinic but optimal fiber placement and light dosimetry remain topic of research. It is attempted to create devices that are capable of real-time measuring light and PS distribution, so appropriate dosimetry can be calculated and adjusted automatically during the intervention [315].

Tumor hypoxia is an obstacle for many cancer therapies. In PDT this is even more pronounced as the photodynamic effect depends heavily on the presence of molecular oxygen while the production of ROS itself depletes tissue oxygen levels as well. Simple solutions such as hyperbaric oxygen or fractionated PDT have some effect but results vary. Pre-PDT existing hypoxia can be circumvented by PSs that are able to produce radicals other than ROS in the absence of oxygen. It is also possible to take advantage of hypoxia by co-delivering drugs that are activated under low oxygen conditions. Otherwise, PDT induced hypoxia can be circumvented by conjugating compounds that are able to capture singlet oxygen during irradiation and releasing it in the dark phase of fractionated PDT. This way tissue re-oxygenation can take place whilst still damaging tumor tissues.

Besides trying to overcome the currently existing limitations of PDT in the clinic, other research is focussed on improving certain aspects to stimulate the applicability of PDT as a treatment modality. Combining the therapeutic property of PDT with diagnostics will not only expand its application but will also improve treatments as immediate evaluation of treatment outcome is possible and follow-up can be adjusted accordingly. Theranostics are often built around nanocarriers which can also be used to implement other therapeutics to create multifunctional agents capable of diagnosing disease and simultaneously treat it with different therapeutics. The combination of PDT with other therapeutics is an exciting prospect that could lead to enhanced efficacy and better applicability in the clinic.

Another exciting aspect is the immune reaction following PDT-induced tumor cell death. Considerable amounts of preclinical data and a few clinical reports underline the importance of the immune system in PDT efficacy [366]. With preclinical studies, more light is shed on the immunological events following PDT and their effect on treatment outcome. With sufficient knowledge, this aspect can theoretically be used to make PDT a systemic modality capable of targeting both local and distant tumors. Moreover, cancer vaccines based on cell lysates created by PDT treated tumor cells prove to be effective in establishing rejection of tumor challenge, inhibiting tumor growth or even shrinking tumors. In the future, optimal use of the PDT associated immune response and anti-tumor memory might lead to effective cancer vaccines and the applicability of PDT for metastasizing tumors.

## 7. Conclusions

Based on this review, we believe that PDT deserves a more central position in cancer treatment, either as part of a multimodal approach or as standalone treatment for early disease, palliative care or salvage treatment. With the discovery of new PSs and nanobased formulations, current limitations can be overcome and together with established and improved protocols, better equipment and improved dosimetry can make PDT a strong alternative for conventional treatments. Clinical studies show its enormous potential while preclinical studies indicate there still is room for improvement on multiple fronts.

## Figures and Tables

**Table 1:** Overview of clinically approved PSs.

PS	Excitation Wavelength	Approved	Indication
porfimer sodium/Photofrin^®^	630 nm	Worldwide, withdrawn in EU for commercial reasons	High grade dysplasia in Barret’s Esophagous. Obstructive esophageal or lung cancer
5-ALA/Ameluz^®^/Levulan^®^	635 nm	Worldwide	Mild to moderate actinic keratosis
Metvix^®^/Metvixia^®^	570–670 nm	Worldwide	Non-hyperkeratotic actinic keratosis and basal cell carcinoma
temoporfin/mTHPC/Foscan^®^	652 nm	Europe	Advanced Head and neck cancer
talaporfin/NPe6/Laserphyrin^®^	664 nm	Japan	Early centrally located lung cancer
verteporfin/Visudyne^®^	690 nm	Worldwide	Age-related macular degeneration
Synthetic hypericin/SGX301	570–650 nm	Orphan status in EU	Cutaneous T-cell lymphoma
Redaporfin^®^/LUZ11	749 nm	Orphan status in EU	Biliary tract cancer

## Supplementary Materials

The following are available online at http://www.mdpi.com/2072-6694/9/2/19/s1. Table S1: Summary of clinical studies reviewed.
