# Supplementary material for: Oncologic Photodynamic Therapy: Basic Principles, Current Clinical Status and Future Directions
Source: Cancers (Basel). 2017 Feb 18;9(2):19. doi: 10.3390/cancers9020019 (PMC5332942; doi:10.3390/cancers9020019)
Supplement: Supplementary file 1 [file cancers-09-00019-s001.pdf]

Supplementary Materials: Oncologic Photodynamic Therapy: Basic Principles, Current Clinical Status and Future Directions

Demian van Straten, Vida Mashayekhi, Henriette S. de Bruijn, Sabrina Oliveira and Dominic J. Robinson

Table S1. Summary of clinical studies reviewed.

Lung	Study Goal	Study Method	PS	Study Outcome	AEs	Reference
Endobronchial non-small cell lung cancer	Palliative treatment	Multimodal PDT with HDR (*n* = 9)	Photofrin®	Prolonged local tumor control in 89%	Bronchial contraction	[132]
Advanced non small cell lung cancer with central airway obstruction	Palliative treatment	Multimodal PDT with CT, RT or CRT (*n* = 10)	talaporfin (NPe6)	CR 20%, PR 70%, NR 10%; Mean FEV1 1.70 ± 0.69 L before and 1.99 ± 0.60 L after PDT ($p = 0.029$); 1-year survival 70% with PDT vs. 35% with CT as stated in literature	Mild itching	[133]
Stage IIA-IV intractable non small cell lung cancer	Palliative treatment	Multimodal PDT with CT (*n* = 12)	talaporfin (NPe6)	Median stenosis rate pre-PDT was 60%, one week post-PDT 15% ($p = 0.0003$) and one month post-PDT 15% ($p = 0.0016$); PDT improved FVC (1.93 vs. 2.58 L) and FEV (1.28 vs. 1.67 L); Improved QoL and opening of bronchial lumen in all patients	No PDT related morbidity or mortality	[135]
Stage IIIA and IIIB central non small cell lung cancer	Improve tumor resectability with PDT	Pre-operative CT in some cases PDT (*n* = 42)	talaporfin (NPe6)	CR 33% after CT + PDT vs. 10% after CT ($p = 0.054$); Radical resection in 89% of operable patients after CT + PDT vs. 54% after CT ($p = 0.038$)	Hemoptysis	[136]
Lung cancer or thymoma with pleural spread	Postoperative treatment	Postoperative PDT vs. standard care (CT or RT) (*n* = 18)	Photofrin®	3- and 5-year survival rate were 68.9% and 57.4% respectively. Median OS of 39 months after PDT vs. 17.6 months after CT or RT ($p = 0.047$)	Erythema, air leakage from lungs	[137]
Stage II-IV intractable bronchial lung cancer with lumen obstruction	Palliative treatment	Standalone PDT (*n* = 30)	Photofrin®	OR 86.7% with CR 13%, PR 73%, MR 13%. Mean obstruction % decreased from 90% to 16.7%	Increased phlegm, secretions, mild pain, photosensitivity with local redness, swelling and scurf	[139]
Centrally located early lung cancer with lesions < or >1 cm	First-line treatment	Standalone PDT for lesions > or < 1 cm (*n* = 75)	talaporfin (NPe6)	CR in 94% of lesions ≤1 cm and 90.4% of lesions ≥1 cm ($p = 0.368$); NPe6 proved more effective for lesions >1 cm compared to Photofrin® in earlier studies	Not reported	[142]

Multiple primary lung cancers	First-line treatment	PDT alone or combined with surgery (*n* = 39)	talaporfin (NPe6)	CR was 100% for lesions treated with PDT alone or with surgery + PDT	Not reported, no skin photosensitivity after 2 weeks	[143]
Carcinoma in situ and microinvasive carcinoma of the central airways	First-line treatment	Light-dose ranging study (*n* = 17)	HPPH	Overall response after 1-month CR 82.4%, NR 17.6%. After 6-months CR 72.7%	Mucus plugs, coughing, mild pain, erythema, photofobia	[145]
Malignant pleural mesothelioma	Retrospective study of multimodal treatment	Lung sparing operation followed by intraoperative PDT (*n* = 38) Median FU 34.4 months	porfimer sodium	37/38 (97%) Macroscopic complete resection of stage III/IV using lung sparing surgery Median survival 31.7 months, Median progression-free survival 9.6 months	Not reported	[138]
**Esophagus**	**Study Goal**	**Study Method**	**PS**	**Study Outcome**	**AEs**	**Reference**
High grade dysplasia in Barrett's esophagus, adenocarcinoma and squamous cell carcinoma	First-line treatment	Standalone PDT or combined with adjuvant RT and CT (*n* = 50)	Photofrin®	CR without recurrence was seen in 32%, 30% alive with recurrence, 38% died of recurrence or other causes	Esophagal strictures, mild photosensitivity and pleural effusions.	[152]
Barret's esophagus with high grade dysplasia, intramucosal carcinoma, or T1 cancer	Palliative treatment	PDT with continued use of proton pump inhibitor (*n* = 116)	Photofrin®	At 12 months post-PDT, 70% was HGD and carcinoma free. Complete ablation of BE was achieved in 39%	Not reported	[153]
High grade dysplasia in Barrett's esophagus and mucosal carcinoma	First-line treatment	Light and PS dose-ranging study (*n* = 11)	Photofrin®	CR was seen in 45%, 45.5% had recurrence and 9% was undertreated with residual disease	Esophagal strictures	[154]
High grade dysplasia in Barrett's esophagus and mucosal carcinoma	First-line treatment	PDT with continued use of proton pump inhibitor and EMR (*n* = 126)	Photofrin®	Three months after PDT, 40% had no or nondysplastic BE and 60% were considered non-responders	Not reported	[155]
High grade dysplasia in Barrett's esophagus and mucosal carcinoma	First-line treatment	PDT with EMR (*n* = 31)	Photofrin®	No evidence of HGD was seen in 77% during follow up. Of this group, 8.3% had recurrence 9 months post PDT	Not reported	[156]
Advanced obstructing esophageal cancer	Palliative treatment	Multimodal PDT with RT and stenting (*n* = 20)	Photofrin®	Improved dysphagia scores were reported in 90% of patients. Median OS was 7 months with one patient achieving CR lasting 28 months post-PDT	Esophageal stricture (*n* = 2), skin pigmentation (*n* = 3), and facial edema (*n* = 1)	[157]
High grade dysplasia in Barrett's esophagus	First-line treatment	PDT with continued use of proton pump inhibitor and EMR (*n* = 129)	Photofrin®or HPD	After 1 and 3 years post PDT, CR was 88% and *6% respectively. Of initially failed responses, 70% achieved CR following retreatment. Carcinoma developed in 6.2%	Strictures (27%), Photosensitivity (60%), blistering (7%)	[161]

High grade dysplasia in Barrett's esophagus	Salvage treatment	Standalone PDT (n = 21)	Photofrin®	Of 19 patients included in the follow-up, CR was achieved in 16 with a 5-year DSF of 84%. Significant reduction in the length of Barrett's segment post PDT (p = 0.035)	Photosensitivity (n = 2), strictures (n = 7), chest pain, vomiting	[162]
High grade dysplasia in Barrett's esophagus and adenocarcinoma	First-line treatment	Standalone PDT (n = 125)	Photofrin®	CR was 71.9% of the PDT group. Cumulative occurrence of recurrence was 49.7%	Photosensitivity (10.4%), strictures (10.4%), nausea and vomiting	[163]
High grade dysplasia in Barrett's esophagus	First-line treatment	Dose-ranging study PDT with EMR (n = 16)	5-ALA	For 30 mg/kg ALA, 25% achieved succesful treatment compared to 100% of 60 mg/kg group with red light and 16.6% for green light. PD was 4% in the optimal dose group	Mild photosensitivity (n = 1), gastrointestinal bleeding (n = 2), odynophagia and chest discomfort (40%) Nausea and vomiting, pneumonia (n = 4)	[164]
High grade dysplasia in Barrett's esophagus	First-line treatment	Standalone PDT (n = 64)	5-ALA or Photofrin®	CR achieved in 16/34 (47 %) and 12/30 (40 %) for 5-ALA and Photofrin® respectively (p = 0.34). For lesions <6 cm ALA-5 performed better (p = 0.02) but no difference was seen for bigger lesions (p = 0.37)	AE after Photofrin® vs. 5-ALA: strictures (33% vs. 9%) and photosensitivity (43% vs. 6%)	[165]
High grade dysplasia or early intramucosal adenocarcinoma of esophagus	First-line treatment	Light- and drug-dose ranging study (n = 36)	HPPH	Initial CR was 100% with optimal dosage. The 1-year CR rate across all treatment regimens was 72% (13/18). Of 13 patients with CR, 7 (54%) did not show recurrence on follow-up at 5 years	Photosensitivity, mild to moderate chest pain with one case of severe pain, dysphagia, nausea, odynophagia, strictures (n = 3), pleural effusions(n = 4)	[166]
Early squamous cell carcinoma without metastasis	Salvage treatment	Standalone PDT (n = 9)	talaporfin (NPe6)	CR achieved by 5 out of 9 patients (55.6%)	Mild fever (n = 1), dysphagia (n = 1), pain (n = 3)	[167]
Early squamous cell carcinoma without metastasis	Salvage treatment	Standalone PDT (n = 25)	Photofrin®	CR in 19 of 25 patients (76%), 3-year PFS and OS rates were 40% and 38.4% respectively	Chest pain (n = 15), pharyngeal pain (n = 4), dysplasia (n = 10), mild fever (12) and photosensitivity (n = 8), stenosis requiring dilatation (n = 6), death by aortic-esophageal fistula (n = 1)	[168]

Skin	Study Goal	Study Method	PS	Study Outcome	AEs	Reference
Carcinomas of the breast, colon, prostate, squamous cell, basal cell, and endometrium; malignant melanoma; mycosis fungoides; chondrosarcoma; and angiosarcoma	Explorative	Explorative (*n* = 113)	HPD	111 out of 113 responding complete or partial<br>All types are responding	Prolonged skin photosensitivity	[12]
NMSC: BCC, AK, Bowens, SCC	Review		Photofrin®, ALA, Metvix®, Levulan®,	BCC (n + s): 85% with Photofrin®<br>sBCC: 92% with ALA<br>nBCC: 71% with ALA<br>AK: 75–90% levulan<br>Bowens: 95% CR at 10 months Photofrin®, 90% at 15 months ALA<br>SCC: > 50% ALA<br>(compiled data of 14 studies)		[171]
BCC, SCC, nodules of carcinoma of the breast, AK	Explorative	Explorative	ALA	BCC (*n* = 80): 90% CR, 7.5% PR, 2.5% NR, 2–3 months FU<br>In-situ or early invasive SCC (*n* = 6): All CR<br>SCC >10 mm (*n* = 2): PR<br>AK (*n* = 9): 9 CR	Not reported	[172]
NMSC	Review	Different pretreatments	ALA and MAL	Simple pre-treatments or additions to the regular practice of PDT improve clinical outcome	Not reported	[173]
nBCC	Curative treatment	Debulking vs. no debulking<br>8 weeks FU histo<br>Pain visual analougue scale (*n* = 43)	ALA vs. MAL	*n* = 22 vs. 21<br>No difference both groups had 6 residual tumor<br>Costs of MAL 6 times higher	Not reported	[176]
AK, BD, nBCC, sBCC	Curative treatment	Gentle (AK, BD, sBCC) or debulking curettage (nBCC) 3 h cream, ill aktilite 37 J/cm^2<br>Repeated within few weeks for BD, sBCC, nBCC (*n* = 203 in 116 patients)	ALA vs. MAL	ALA vs. MAL: %CR (n)<br>AK: 63% (24) vs. 75% (44)<br>BD: 89% (9) vs. 78% (18)<br>nBCC: 84% (19) vs. 84% (25)<br>sBCC:88% (25) vs. 87% (39)	Not reported	[177]

sBCC	Multicentre, randomised, controlled, open study	PDT vs. surgery<br>MAL: 2 sessions, 7 days apart, repeated after 3 months if needed or surgery<br>3 h cream, ill aktilite<br>37 J/cm^2<br>(*n* = 196 patients, 1.4 lesions/paient)<br>12 months FU	MAL	CR after 3 months<br>92.2 % MAL, 99.2% Surgery<br>Recurrence after 12 months<br>9.3% MAL, 0 Surgery<br>CO excellent/good<br>94,1% MAL, 59,8% surgery	MAL: 37/100 65 related AE<br>Surgery: 14/96 21 related AE<br>Most AE were expected of dermatological nature and all were mild or moderate<br>21 serious AE non related	[178]
sBCC	Single blind, non inferiority, randomizsed controlled trial	PDT vs. Imiquimod vs. Fluorouracil<br>(*n* = 583 patients)<br>12 months FU	MAL	26 lost to FU<br>Recurrence-residue:<br>MAL: 52/196<br>Imi: 31/189<br>5 fu:39/198<br>Imi vs. MAL p = 0.021<br>CO (good-excell): 62.4% MAL, 61.4% imi, 57.5% 5-FU, no diff.<br>Compliance: 100% Mal, 20.9% imi, 31.3% 5-FU	MAL: no AE<br>Imiquimod: 1 patient<br>Fluorouracil: 2 patients<br>Local wound infection	[179]
sBCC	Randomized prospective trial	Single vs. light fractionation<br>(*n* = 745)<br>5 year FU	ALA	88.4%CR fractionated PDT<br>75.4%CR Single ill	Not reported	[180]
sBCC	Explorative:<br>Pain reduction through low-high fluence rate illuminations	Low fluence rate illumination until photobleaching reached to 90% followed by high FR ill till 200 J/cm^2<br>(*n* = 33 in 26 patients)	ALA	Fluence to 80% fluorescence at 10, 20, 40, 50, 60 and 150 mW/cm^2 was 5.7, 4.5, 7.5, 7.4, 12.4, and 28.7<br>No sign pain with FR < 50 mw/cm^2<br>CR comparable to continuous treatment	Not reported	[181]
sBCC	Prospective study on pain control	Low fluence rate illumination until photobleaching reached to 90% followed by high FR ill till 200 J/cm^2<br>(*n* = 25 sBCC)<br>FU to 24 months	MAL	First 40 or 50 or35 mW/cm^2 than 70 mW/cm^2 to 75 J/cm^2<br>Pain increased with increasing irradiance. And cr decreased.	Not reported	[182]

AK	Randomized, double-blind, prospective study	Split-scalp curative comparative study<br>Pain assessment<br>(n = 15)<br>FU 1 months	ALA 5 h vs. MAL 3 h	Reduction of lesions<br>6.2 ± 1.9 ALA<br>5.6 ± 3.2 MAL<br>No sign diff.<br>Sign. More pain with ALA<br>Pt preference MAL		[183]
AK on the scalp	Comparative study	Waldman 100 J/cm² at 160 mW/cm²<br>(n = 69)	ALA vs. MAL	54% discontinued ALA treatment<br>14% discontinued MAL treatment		[184]
AK	Multicentre, randomised, observer-blind phase III trial	PDT vs. placebo<br>(n = 571)<br>3 months FU<br>Max 2 times PDT 3 months apart	BF-200 ALA vs. MAL	CR BF-200 ALA: 78.2%<br>Plac.: 17.1% $p < 0.0001$<br>MAL:64.2% $p < 0.05$<br>Lesion CR, BF: 90.4%<br>MAL: 83.2%, Placebo.: 37.1%<br>Analysis of subgroups (grade, target areas) showed sign better BF-200 than MAL/Placebo	No difference in related EA MAL vs. BF-200 ALA (application site erythema, burning and pain)<br>Narrow spectrum devices—more and worse AE	[185]
AK	Multicentre, randomised, observer-blind phase III trial	FU of 2 phase II studies<br>(n = 630)<br>6 en 12 months FU	BF-200 ALA vs. MAL	Proportion that remains completely clear: (in favour of)<br>47% BF-200 vs. 36% MAL<br>No safety concerns	5 related AE in (patients):<br>SCC (1), BF-200 ALA (2), BCC (2), MAL (1) Bowens (1) MAL, Placebo (1)	[186]
AK	Retrospective monocentric Study Pain	Superficial curettage, Ibuprofen, 3 h cream, ill aktilite 37 J/cm²<br>VAS pain score during and 8 h post (n = 173 patients, 965 lesions)	MAL vs. BF-200	MAL vs. BF-200 during VAS score: 5 vs. 5.8 $p < 0.001$<br>Treat. Interupt: 13.2 vs. 19.9% $p < 0.001$<br>Severe pain: 25 vs. 36% $p < 0.05$<br>No difference at 8 h	Not reported	[187]
AK	Randomized, double blind, prospective study	Split-face<br>Grade I: 1 PDT session<br>Grade II-III: 2 PDT sessions (n = 13 patients, 177 AK)<br>3 months FU	BF-200 ALA vs. MAL	BF vs. MAL<br>Complet clearance rate 84.5% vs. 74.2% $p = 0.099$<br>No difference in reduction grading $p = 0.065$<br>Per patient half-face analysis BF sign better CR grade I $p = 0.027$<br>No preference	Nearly painless and no difference in adverse reactions	[188]

Head and Neck	Study Goal	Study Method	PS	Study Outcome	AEs	Reference
Leukoplakia or erythroplakia	First-line treatment	Standalone PDT (n = 147)	temoporfin or 5-ALA	CR was achieved in 77.6% after first treatment. At the 5-year follow-up CR was 78.9%. At time of writing CR was 81% while 7.5% had PD, 3.4% SD and 8.2% PR	Mild to moderate pain and skin photosensitivity	[201]
Oral squamous cell carcinoma	First-line treatment	Standalone PDT (n = 38); repeated when disease reoccurred 6–7 months after 1st PDT	temoporfin	CR was 68.4% at last review. Overall recurrence of the disease was 15.8% (6/38); and 5-year survival was 84.2%.	Pain, photosensitivity with six patients who failed to avoid direct sun exposure having 1st (n = 2), 2nd (n = 3) and 3rd (n = 1) degree sunburns	[202]
Local persistant and recurrent nasopharyngeal carcinoma	Salvage treatment	Standalone PDT (n = 21)	temoporfin	CR was 95% 10 weeks post-PDT. The unresponsive patient 27 months after initial PDT. Two year PFS was 49% with regional relapse (n = 6) and distant metastasis (n = 5) observed. Mean two year OS was 65%.	Local pain	[208]
Early head and neck squamous cell carcinoma	First-line treatment	PDT vs. Surgery (n = 243)	temoporfin	CR of 86% for T1 and 63% for T2 tumors after PDT (p = 0.005) compared to 76 % for T1 and 79% for T2 after surgery (p = 0.75). LDFS after PDT was 102.6 and 113.8 months for T1 and T2 tumors respectively compared to surgery with 152.7 and 152.8 months for T1 and T2 tumors respectively. Mean OS was 101.5 and 116.9 months for PDT treated T1 and T2 tumors respectively (p = 0.842). After surgery mean OS was 122.6 months for T1 and 109.5 months for T2 tumors (p = 0.450)	Not discussed	[207]
Early head and neck cancer	First-line treatment	PDT vs. Surgery (n = 98)	temoporfin	After one intervention, rate of local control was 89% (49/55) after PDT and 74% (32/43) after surgery (p = 0.07). Including subsequent interventions, 5 year local DFS was 67% and 74% for PDT and surgery respectively. Overall 5 year DFS was 47% for PDT and 53% for surgery. Five year OS was 83% for PDT and 75% for surgery	Not discussed	[206]

Oral squamous cell carcinoma or mucosal dysplasia	First-line treatment	Standalone PDT (*n* = 25)	Photofrin®	Of 18 OSCC patients, 17 achieved CR and 1 PR. CR was 100% for dysplasia patients. Recurrence was observed in both the OSCC (*n* = 2) and dysplasia (*n* = 1) group. Disease specific survival was 95.8%	Swelling, oedema, (sometimes severe) pain, phototoxic skin reactions, skin discoloration	[194]
Early head and neck squamous cell carcinoma and dysplasia	First-line treatment	Light-dose ranging study (*n* = 40)	HPPH	Only the 140 J/cm² group was used for response ratios; CR for dysplasia was 46% and for OSCC 82% ($p = 0.056$) although dysplasia responses were less durable	Pain and oedema (100%), grade III oedema, mild sunburns (*n* = 4)	[197]
T1 Oropharynx	First clinical study	48 h drug-light 100 J/cm² at 100 mW/cm² (*n* = 42 patients) 2 months biopsy	HPD	CR 40 (95%) Recurrence 1 Residual 1	Not reported	[192]
Various cancers	Explorative	Explorative 1 year FU	mTHPC PII (1 patient) ALA (1 patient)	Good clinical responses		[195]
Tis-2, face, oropharynx, larynx	Prospective clinical study	48 h drug-light 100 J/cm² at 100 mW/cm² (*n* = 83 patients) 2 months biopsy	Photosan III®	51 of 57 BCC, 6 of 7 SCC skin, 6 of 7 Oro CA 11 of 12 larynx CA Complete histological FU 13–71 months	Not reported	[193]
Carcinoma of the lip SCC	Non-randomized phase II study	0.15 mg/kg 96 h drug-light 20 J/cm² at 100 mW/cm² (*n* = 25 patients) 12 weeks FU biopsy	Foscan®	24/25 = 96% CR 12 weeks 2 recurrences (4,18 months) Lymphnode meta 7 months CO better than surgery	Swelling, local pain Photosensitivity in 5 patients	[204]
All kinds of cancer Not just H & N	review	review		Endoscopic light delivery Interstitial light in buried tumors that would require extensive surgical resection Adjuvant therapy after removal of bulk tumor		[199]
Oral cavity and oropharynx	Prospective study	0.15 mg/kg 96 h drug-light, nasotracheal intubation ill (*n* = 25 patients-29 T1-T2 NO) 37 months FU	mTHPC	25 (86%) CR 4 recurrent lesions had conventional therapy	No permanent impairment of mastication, swallowing and articulation of speech	[196]

Persistent or recurrent H & N cancer unsuitable for surgery, radiotherapy or chemo	Phase I-II Salvage treatment	0.15 mg/kg 96 h drug-light Interstitial PDT 20 J	mTHPC	45 patients, 9 CR, 5 10–60 months 24 Sympt relief, Median survival 16 months in 33 responders Median surv 2 months in 12 nonresponders	Carotid blow out at 2 weeks post PDT	[200]
Recurrent respiratory papillomatosis Needed surgery every 3 months	Parallel-arm, randomized trial	Single PDT 6 days drug-light 80–100 J adult/60–80 J child (*n* = 15 patients + 2); 12 months FU	mTHPC	5 remission with recurrences at 3–5 years Tracheal disease not responsive No change prevalance latent pap virus DNA	Not reported	[203]
Various deep-seated H & N region upper and lower limbs	Prospective study	0.15 mg/kg (*n* = 68 patients) 7 months FU	mTHPC	2 patients free of disease Half patients good responses A third moderate resp. 6 weeks FU radiological: 13 patients no response, 18 patients minimal, 23 moderate 11 sign. Resp.	A range of AE reported in number not in severity	[209]
Oral SCC and fieldcancerisation	Light dose ranging study	0.15 mg/kg 72–96 h drug-light (*n* = 20)	mTHPC	12*single lesions < = T3 cleared all 6 T4 3 out of 6 cleared T1 + T2: 9 of 14 cleared (field cancerization)	Not reported	[205]
Paranasal sinuses Scc, adenocarcinoma, undiff. Carcinoma, adenoid cystic carcinoma, risarcoma	Adjuvant therapy to surgery	Open and endoscopic approach (*n* = 15)	mTHPC	Macroscopic resection *n* = 3 Debulking surgery *n* = 12 *N* = 5: CR	No AE *N* = 4: Temporary diplopia *N* = 1: ext. necr. Oro-nasal fistula	[213]
Sino-nasal malignancies	Retrospective analysis	PDT (*n* = 7)	mTHPC	No CSF leakage, meningitis or major bleeding. Temporary diplopia in 3 patients, facial edema and pain in all patients		[212]
**Bile Duct**	**Study Goal**	**Study Method**	**PS**	**Study Outcome**	**AEs**	**Reference**
Bile duct cancer	Salvage treatment	Multimodal PDT with adjuvant CT or RT (*n* = 7)	talaporfin (NPe6)	CR without recurrence in 3 of 7 patients. Two deaths by liver metastasis. One cancer-unrelated death and one is still alive with cancer	Biliary stenosis (*n* = 1), transient epithelial or peribiliary inflammation (*n* = 2), mild transient cholangitis (*n* = 1), mild photodermatitis (*n* = 2), skin pigmentation (*n* = 1)	[218]

Bile duct cancer	Palliative treatment	Biliary stenting with PTD (*n* = 170) vs. stenting alone (*n* = 157)	Photofrin®	Increase in length of survival, improvement of Karnofsky scores and decrease of serum bilirubin after PDTtreatment were observed	Biliary sepsis (15%), Skin phototoxicity (6%)	[217]
Bile duct cancer	Palliative treatment	Standalone PDT (*n* = 11)	Foscan®	Median survival was 18 months after PDT. Four patients showed tumoricidal depth of >7.5 mm and prolonged relief of cholestasis and jaundice. QoL improved in 6 of 11 patients.	septic cholangitis, recurrent liver abscess, skin photosensitivity, cholangitis	[220]
Bile duct cancer	Palliative treatment	PDT plus stenting (*n* = 29)	Foscan®	Median time to local tumor progression was 6.5 months, fewer PDT treatments needed, Median survival time was 15.4 months	cholangitis (*n* = 4), liver abscess (*n* = 2), cholecystitis (*n* = 2), phototoxic skin (*n* = 5), and injection site reactions (*n* = 7)	[221]
Bile duct cancer	Palliative treatment	Endoscopy-guided PDT using porfimer (*n* = 12) and talaporfin (*n* = 13)	Porfimer and talaporfin	Higher cytocidal effect with talaporfin than that of porfimer after PDT. Less Phototoxicity caused by talaporfin and therefore staying at hospital after PDT was shorter	Photosensitivity	[225]
Bile duct cancer	Palliative treatment	Endoscopic ultrasound-guided PDT using Photolon (*n* = 4)	Photolon®	The median volume of necrosis was 4 cm^2, Disease remained stable during a median follow-up of 5 months	No treatment-related mortality or complications	[226]
Unresectable hilar carcinoma	Palliative treatment	PDT combined with stenting (*n* = 13)	Foscan®	Five out 13 patients died within 13 months post-PDT. Eight patients had a median survival time of 13 months and were still in follow-up at time of writing the paper	Abdominal pain, nausea	[222]
Unresectable hilar carcinoma	Palliative treatment	PDT + CT vs. PDT alone (*n* = 43)	Photofrin®	Median OS of 8 months for PDT vs. 17 months PDT+S-1 arm. One-year survival was 32% and 76.2% respectively. PFS was 2 months vs. 10 months respectively	Liver abscess (*n* = 3), photosensitivity (*n* = 4), cholangitis (*n* = 4)	[223]
Unresectable hilar carcinoma	Palliative treatment	PDT + CT vs. PDT alone (*n* = 68)	Photofrin®	Mean survival time of 374 days for PDT vs. 520 for PDT+CT ($p = 0.021$). The 1-year survival rate was significantly higher in the PDT group (88% vs. 58%, $p = 0.001$) Mean OS was 395 days for PDT vs. 566 days for PDT + CT ($p = 0.09$)	No specifics given.	[224]

Pancreas	Study Goal	Study Method	PS	Study Outcome	AEs	Reference
Locally advanced unresectable pancreatic adenocarcinoma	First-line treatment	Light dose finding study possibly followed by CT or RT (*n* = 15)	verteporfin	SD was achieved in 11/13 assessable patients and PD in 2/13, 1 month post-PDT. In 6 patients SD maintained for 3 months. Median OS was 8.8 months	Mild to moderate abdominal pain, mild inflammation	[232]
**Bladder**	**Study Goal**	**Study Method**	**PS**	**Study Outcome**	**AEs**	**Reference**
Intermediate or high-risk urothelial cell carcinoma	Postoperative PDT	Surgery followed by PDT (*n* = 17)	HAL	Post-PDT CR rate was 52.9%, 23.5% and 11.8% after 6, 9 and 21 months respectively	Mild or severe irritative bladder/urgency syndrome, urinary tract infections	[235]
Recurrent, high grade nonmusclar invasive bladder cancer	Postoperative PDT	Surgery followed by PDT (*n* = 35)	Radachlorin®	Average follow up was 26.74 ± 6.34 months. The recurrence free rate was 90.9% at 12 months, 64.4% at 24 months and 60.1% at 30 months	No severe adverse effects was detected after PDT treatment	[234]
**Female Reproductive Tract**	**Study Goal**	**Study Method**	**PS**	**Study Outcome**	**AEs**	**Reference**
Cervical intraepithelial neoplasia 1–3	Fluorescence diagnosis	Dose-finding study (*n* = 24) fluorescence only	HAL	Fluorescence intensity increased over time with higher values for 10 mM dose than 4 mM, CIN more fluorescent than normal epithelium		[242]
Cervical intraepithelial neoplasia, human papillomavirus infection	First-line treatment	Topical application of PS and illumination 3–5 h later	HAL	15/24 patients had complete response; remission rates of 71, 50 and 71% for CIN 1, 2 and 3	No systemic adverse effects, no cutaneous toxicity. Some patients reported cervical tenderness during illumination	[243]
Cervical intraepithelial neoplasia 2 and 3, with human papillomavirus infection	First-line treatment	PDT repeated with 1 week interval	ALA	CIN2 with complete response for 9 months, one CIN3 remained positive for 6 months after 4 treatments	Burning sensation and increased vaginal discharge	[238]
Cervical intraepithelial neoplasia 2 and 3	First-line treatment	Standalone treatment	Photolon® (chlorin e6 derivative)	104 of 112 (CIN2: 24, CIN3: 88) women had complete response	Pain during treatment, increased body temperature, several patients with mild arterial hypertension after PDT	[239]
Cervical intraepithelial neoplasia 1–3	First-line treatment	Double-blinded dose finding study	HAL or MAL	From initial CIN1 (*n* = 3), CIN2 (*n* = 9), CIN3 (*n* = 13), 9 patients had a complete response, 7 had a partial response 6 months after PDT. No macroscopic changes of the cervix observed	No systemic effects were observed	[247]

Cervical intraepithelial neoplasia 1–3	First-line treatment	Light- and drug-dose ranging study (n = 67)	MAL or HAL	A 3 h DLI had the best results with CR rates of 50% for MAL (1.2 M), 33% for HAL (10 mM) and 46% for HAL (40 mM). HAL40 with 25 J/cm2 light achieved 29% CR compared to 33% with 50 J/cm2. HAL40 with 3 h DLI followed by a light dose of 50–100 J/cm^2 was most effective	cervical pain and spasms and cervical or vaginal discharge	[244]
Cervical intraepithelial neoplasia 1	First-line treatment	PDT vs. Placebo or follow-up only (n = 70)	HAL	HAL: CR of 57% (n = 47) Placebo or follow-up only: CR of 25% (n = 23) (p = 0.04)	Mild to moderate local pain, cramping and vaginal discharge	[245]
Cervical intraepithelial neoplasia 1–2	First-line treatment	Dose-finding study (n = 262)	HAL	No significant difference in response between CIN1/2 patients receiving PDT or placebo. For CIN2 patients alone, only HAL 5% had a statistically significant efficacy compared to placebo with CR in 18/19 (p = 0.009) after 3 months and 18/19 (p = 0.021) after 6 months. HPV clearance was not significantly higher	Pelvic pain, vulvovaginal discomfort, vulvovaginal burning sensation, vulvovaginal pain, genital discomfort, abdominal pain, upper abdominal pain, procedural pain, and postprocedural discomfort	[246]
Peritoneal Carcinmatosis and sarcomatosis	Phase II study	2.5 mg/kg, 48 h drug-light, debulking surgery, intraoperative ill (n = 100 patients)	Photofrin®	No sign. CR or long-term tumor control	Capillary leak syndrome, 2 postoperative deads, 1 intra-abdominal bleeding, 4 adult respiratory distress syndrome, 4 bowel fistulae or anastomica leak, 2 wound dehiscence or delayed wound healing, 2 wound infections, 3 prolonged ileus or small bowel obstruction, reversible abnormal liver function, hypocalcemia and hypomagnesemia, 20 mild photosensitivity	[237]
Premalignant lesions of vulva and vagina	Retrospective study	Colposcopy and or vulvoscopy, surface PDT 2 mg/kg, 48 h drug-light, 150 J·cm^{-2} (n = 15 patients) 1 year FU	Photogem®	3 months FU: 80% (12/15) CR, 1-year FU: 71.4% (10/14) CR	13.3 % (2/15) Facial edema and urticaria 1/15 perineal pain	[241]

Vulvar HSIL (formally denoted vulvar intraepithelial neoplasia)	Retrospective study	Evaluation of 3 treatment modalities: CO2 laser vaporation, PDT, excision/ vulvectomy (*n* = 93) Mean FU 53.7 months	ALA	Recurrences CO2: 40.4% PDT: 48.1% Excision: 42%		[240]
**Prostate**	**Study Goal**	**Study Method**	**PS**	**Study Outcome**	**AEs**	**Reference**
Early diagnosed prostate cancer	First-line treatment	Dose-finding study and PDT (*n* = 6)	mTHPC	67% decrease in prostate specific antigen (PSA) level, Oedema and patchy necrosis shown in MRI scans	No specifics given	[250]
Localised prostate cancer	Pre-operative PDT	PDT followed by surgery (*n* = 19)	5-ALA	Selectively accumulation of 5-ALA in cancer cells, No PpIX enrichment in benign tissue, stroma (0/19)	Cutaneous phototoxicity	[249]
Recurrent prostate cancer	Salvage treatment	VTP after failure of external beam radiation therapy (*n* = 24)	Tookad®	Regions of Avasculrity with no viable tumor observed in MRI images at 7 days but outside this zone, viable tumor was remained, in all patients In 4 patients, PSA decreased to negligible level	Self-limited hypotension after drug infusion	[251]
Recurrent prostate cancer	Salvage treatment	VTP after failure of external beam radiation therapy (*n* = 28)	Tookad®	8/16 patients treated with high light dose at least 23 J/cm² in 90% of prostate volume were biopsy negative at 6 months. PSA level decreased to negligible level in this group of 8 patients	Initial deterioration in voiding fuction which was controllable, rectourethral fistulae in 2 patients	[252]
Localised prostate cancer	First-line treatment	Dose-finding study and VTP (*n* = 84)	Tookad®	At 6 months, 74% of patients were biopsy negative biopsy, Mean percentage of prostate necrosis at 7 days after VTP was 78%	No specifics given	[253]

Brain	Study Goal	Study Method	PS	Study Outcome	AEs	Reference
Malignant glioma	FGR	FGR (*n* = 139) vs. conventional surgery (*n* = 131)	ALA	Contrast-enhancing tumor completely ressected in 90% of 139 patients, compared to 47% in 131 patients; 6 months extended progression free survival with FGR	Most frequent early AE: hemiparesis (4 vs. 2); aphasia (3 vs. 0); convulsions (3 vs. 1); and epidural haematoma (1 vs. 1)	[255]
Malignant gliomas, metastatic brain tumors, meningiomas	Newly diagnosed or recurrent tumor (not in randomized trial)	PDT (*n* = 112) post-resection	Photofrin®	Overall survival 42 weeks (*n* = 96)	3 deaths post-PDT; 7 patients increased neurological defect, 2 recovered within 1 month; 4 patients deep vein thrombosis; 75% no complications	[258]
Malignant brain tumors	FGR followed by intra-operative PDT	FGR + PDT vs. control	Foscan®	Tumor prediction with 90.7% accuracy; 75% radical resection with FGR, compared to 52% without FGR; mean survival of 9 months for FGR+PDT, compared to 3.5 months	2 severe toxic responses to sunlight; 1 transitional brain swelling	[259]
Malignant glioma	PPIX selectivity (FGR) and bleaching (PDT)	FGR vs. surgery; followed by PDT	ALA	Mean PpIX fluorescence 100-fold increased in viable tumor; PpIX bleached 8, 16 and 1% with 100, 150 and 200 J/cm2, respectively; Complete resection in 65% patients with FGR, compared to 36%		[256]
Glioblastoma multiforme	FGR and repetitive PDT	FGR after surgery and 5 illumination sessions for PDT (*n* = 13) vs. surgery alone (*n* = 14)	ALA, Photofrin®	Mean survival 52.8 weeks compared to 24.6 weeks; mean time to tumor progession 8.6 months, compared to 4.8 months	3 incidents of deep venous thrombosis, 2 in the study group; no other AE described	[257]
Unresectable gliomas	Intraoperative treatment	Intraoperative PDT often followed by CRT (*n* = 14)	talaporfin (NPe6)	Newly diagnosed gliomas: CR 1/6, PR 2/6, DP 1/6, not evaluable 1/6. Median OS was 26 months. Recurrent gliomas: CR 1/8, PR 1/8, SD 2/8, PD 4/8. Median OS was 9 months. All patients had (second) recurrence	Hemiparesis and brain oedema but neither directly PDT related	[260]
Primary malignant parenchymal brain tumors	Intraoperative treatment	Multimodal. Surgery and PDT (*n* = 22)	talaporfin (NPe6)	All gliomas: median PFS of 20 months, median LPFS of 22.5 months and a median OS of 27.9 months. Newly diagnosed giomas: median PFS of 12 months, median local PFS of 20 months and OS was 24.8 months	Mild rash, blister, redness of the skin	[261]

Other Organs	Study Goal	Study Method	PS	Study Outcome	AEs	Reference
Gastric cancer	Case report	1 patient inoperable gastric cancer, PDT over 2 years, once with Photofrin®, three times with talaporfin sodium	Photofrin® or talaporfin sodium	Repeated PDT treatments lets patient digest food in natural way	Transient examples of slight anemia and hypoalbuminemia, Photofrin®: slight tanning, slight abdominal pain and diarrhea	[263]
AIN grade IIII	Prospective intervention study	PDT 0.075–0.15 mg/kg, 48 h drug-light, red or green light (*n* = 15 patients and 25 PDT sessions)	mTHPC	28% (7/25) initial CR, 16% (4/25) initial PR, 8 months FU: 7/11 recurrences, Green PDT: 6–15 months FU: 16% (4/25)	Intensive pain, bloody and purulent rectal discharge. anal stricture formation in one patient	[264]
AIN III, AIN I or II, Paget's disease	Retrospective study	Photofrin®: 1.2 mg/kg 630 nm light 100 J·cm^{-2} 400 mW cm-2, ALA: 37.5 J·cm^{-2}, 2 cycles 3–5 minutes dark interval (*n* = 15 and 26 PDT sessions) Median FU 19 months	ALA and Photofrin®	ALA: 13 patients<br>Photofrin® 1 patient<br>Both: 1 patient<br>10 patients result: 6 initial CR with AIN II or III, with 3 recurrences at 7, 8 and 22 months 3 PR, resolution of symptoms in 4 patients	1 patient reported significant discomfort	[265]
Circumscribed choroidal hemangioma	Prospective, multicenter, nonrandomized, clinical trial	6 mg m^{-2}, 689 nm, 15 min drug-light, 50 J·cm^{-2}, 83 s, 1–4 treatments 12 weeks apart FU > 12 months	verteporfin	69% Visual recovery, Visual acuity increased, Cystoid macular edema regressed, exudative macular detachment disappeared in all but 2, CCH thickness decreased from 3 to 1.7 mm	No severe adverse effect	[266]
Amelanotic choroidal melanoma	Curative study	Repeated PDT till flat or no change, 4–16 mm diameter and 1.3–5.7 mm thick lesions, (*n* = 9 patients, 1 with pigmented portion)	verteporfin	8 apparent CR in 1–14 months, 1 pigmented area remained 2 mm thick, 8 no recurrence FU 34–81 months, 2 local recurrences at 21 and 34 months	No adverse effects	[267]
Choroidal metastasis	Retrospective interventional case series	6 mg m-2, 689 nm, 600 mW cm^{-2} 83 s, 7 mm diameter and 2.9 mm thick lesions, (*n* = 9 CM in 8 eyes) 1 or 2 sessions	verteporfin	2 non responders, 7 complete control with resolution of subretinal fluid and thickness reduction of 39%, 7 improvement or stabilization of vision	1 intraretinal hemorrhage	[268]

Circumscribed choroidal hemangioma	Prospective consecutive 2 centered, noncomparative interventional case series	6 mg m-2, 689 nm, 50 J·cm^{-2} or 100 J·cm^{-2}, 83 s, retreatments in case of persistent exudation, (*n* = 25 subjects) FU > 5 years	verteporfin	22 patients had 1 PDT session to 100 J·cm^{-2} and no recurrences, 3 PDT sessions to 50 J·cm^{-2} followed by one session to 100 J·cm^{-2}, Foveal center thickness decreased from 386.2 to 179.2 µm, Visual acuity improved by 18.5 letters and more than 2 lines in 19 eyes, all showed complete resolution of macular exudation	No treatment-related adverse events or complications identified	[269]
